# Peripheral Blood Cells from Patients with Hodgkin's and Diffuse Large B Cell Lymphomas May Be a Better Source of Candidate Diagnostic miRNAs Than Circulating miRNAs

**DOI:** 10.1155/2021/3212878

**Published:** 2021-02-04

**Authors:** Ewa Paszkiewicz-Kozik, Agnieszka Paziewska, Maria Kulecka, Michalina Dąbrowska, Anna Kluska, Aneta Bałabas, Magdalena Piątkowska, Filip Ambrożkiewicz, Joanna Tajer, Włodzimierz Osiadacz, Joanna Romejko-Jarosińska, Martyna Kotarska, Natalia Żeber-Lubecka, Jakub Karczmarski, Lidia Popławska, Michał Mikula, Piotr Rutkowski, Jan Walewski, Jerzy Ostrowski

**Affiliations:** ^1^Department of Lymphoproliferative Diseases, Maria Sklodowska-Curie National Research Institute of Oncology, Warsaw 02-781, Poland; ^2^Department of Genetics, Maria Sklodowska-Curie National Research Institute of Oncology, 02-781, Poland; ^3^Department of Gastroenterology and Hepatology, Medical Center for Postgraduate Education, Warsaw 02-781, Poland; ^4^Department of Soft Tissue/Bone Sarcoma and Melanoma, Maria Sklodowska-Curie National Research Institute of Oncology, Warsaw 02-781, Poland

## Abstract

Hodgkin lymphoma (HL) and diffuse large B cell lymphoma (DLBCL) represent 15% and 20%, respectively, of all lymphoma types. The aim of this study was to identify and compare circulating serum miRNA (c-miRNA) and peripheral whole blood miRNA (wb-miRNA) profiles in patients with these lymphomas. Serum samples (20 HL, 21 DLBCL, and 30 healthy controls) and whole blood samples (21 HL, 17 DLBCL patients, and 30 healthy controls) were collected at the time of diagnosis. Serum and whole blood were also collected from 18 HL/17 DLBCL and eight HL/nine DLBCL patients, respectively, after treatment. Pairwise comparisons identified 125 c-miRNAs (adjusted *P* value < 0.05) showing significant dysregulation between 30 healthy controls and patients; of these, 47 and 55 differentiated controls from pretherapeutic HL and DLBCL patients, respectively. In addition, 60 and 16 c-miRNAs differentiated controls from posttherapeutic HL and DLBCL, respectively. Pairwise comparisons identified 292 wb-miRNAs (adjusted *P* value < 0.05) showing significant dysregulation between 30 controls and patients; of these, 103 and 169 differentiated controls from pretherapeutic HL and DLBCL, respectively, and 142 and 151 wb-miRNAs differentiated controls from posttherapeutic HL and DLBCL, respectively. Thus, lymphoma-associated miRNAs may be a better source of noninvasive candidate biomarkers than miRNAs in serum. It is unclear whether miRNA alterations in lymphoma cells are similar to those observed in white blood cells.

## 1. Introduction

Lymphomas are a heterogeneous group of malignances that arise from B or T cells; they show varying genetics, pathogenesis, clinical presentations, and responses to treatment, resulting in variable outcomes (even among patients with a similar diagnosis) [1–3]. There are more than 100 different lymphoma types, although most are B cell lymphomas [4]. Of these, Hodgkin lymphoma (HL), in which tumor cells constitute less than 1% of the tumor bulk [1, 4], is one of the most common (representing about 15% of all lymphomas) in young adults. Non-Hodgkin, diffuse large B cell lymphoma (DLBCL), comprising about 20% of all B cell lymphomas, is the most common subtype of aggressive B cell lymphoma in adults [1, 4]. Currently, diagnosis of HL and DLBCL depends on pathological assessment of biopsy material supplemented by genetic examination of contentious tissues. However, when insufficient representative biopsy material is available, precise diagnostic and prognostic molecular biomarkers are required.

MicroRNAs (miRNAs) are short noncoding RNAs that regulate expression of numerous genes by modulating both mRNA stability and its translation into proteins. Each of the approximately 1800 human miRNAs may have regulatory roles in multiple cell signaling pathways, and some mediate and/or correlate with oncogenesis, clinical diagnosis, subtype, and malignancy stage [5]. Since miRNAs can be detected readily in tissues and body fluids, miRNA transcriptomes are particularly attractive as candidate oncological biomarkers. A recent study examined circulating miRNAs, either in whole plasma/serum or within exosomes, and found that several circulating miRNAs were differentially expressed in patients with HL or DLBCL [6]. However, reports do not always agree. For example, a systematic review of 12 articles identified only miRNA-21 as a circulating diagnostic biomarker in DLBCL patients [3]. In addition, another review reported that two or more of six studies identified 35 miRNAs that are significantly deregulated in HL patients and have clinical or prognostic implications [6]. Inconsistent results mean that implementation of circulating miRNA analysis into routine clinical practice is still some way off.

Blood cells suspended in a fluid matrix connect all biological systems within an organism; as such, they constitute the first line of immune defense and are considered a surrogate for “traditional” tissue specimens used for clinical diagnosis. Peripheral blood cells share >80% of the transcriptome with other tissues; indeed, changes in whole blood transcriptomes are associated with a wide range of diseases, including neoplastic disorders [6–8]. For example, expression of miR-9, miR-20a, miR-21, miR-26a, and miR-155 in whole blood from HL patients correlates with ABVD treatment responses [9]. In addition, miRNA-21, miRNA-26b, miRNA-16, and miRNA-155 were differentially expressed in peripheral blood mononuclear cells from patients with hepatitis C who develop hepatocellular carcinoma or non-Hodgkin's lymphoma [10].

The aim of this study was to identify and compare circulating miRNA (c-miRNA) and peripheral whole blood miRNA (wb-miRNA) profiles in samples from patients with HL or DLBCL.

## 2. Materials and Methods

### 2.1. Patients

Two cohorts of lymphoma patients were enrolled in this study. The cohort no. 1 included 20 patients with classical HL and 21 DLBCL patients whose serum samples were collected progressively; the cohort no. 2 included 23 HL and 20 DLBCL whose peripheral blood samples were already deposited at the institutional biobank (Table 1). Of the latter groups, the pretherapeutic samples were available from 21 HL patients and 17 DLBCL patients. All included patients were diagnosed at the Department of Lymphoid Malignancy, Maria Sklodowska-Curie Institute Oncology Centre, according to the 2008 WHO classification [1]. HL patients were treated with induction ABVD (adriamycin, bleomycin, vinblastin, and dacarbazine) chemotherapy and DLBCL patients received RCHOP (rituximab, cyclophosphamide, adriamycin, vincristin, and prednisolone). The number of additional radiotherapy cycles varied according to standard ESMO guidelines and local regulations [11, 12]. Serum or whole blood samples were obtained from the patients before starting induction treatment (the pretherapeutic samples). After the end of the therapy, the serum samples were collected from 18 HL and 17 DLBCL patients of the cohort no. 1, while the whole blood samples were available from 8 HL and 9 DLBCL patients of the cohort no. 2 (the posttherapeutic samples), including only 6 and 6 paired samples from the same patients with HL and DLBCL, respectively.

### 2.2. Compliance with Ethical Standards

The study was approved by the local Bio-Ethics Committee and was conducted in accordance with Good Clinical Practice Guidelines, according to the tenets of the Declaration of Helsinki.

### 2.3. Blood Sample Collection

For c-miRNA analysis, blood samples were collected in Serum Gel S/7.5 ml tubes (Sarstedt S-Monovette) after venipuncture, allowed to clot for 60 min at room temperature, and then centrifuged at 1300 × g for 10 min at 4°C. Next, 500 *μ*l serum was aliquoted into 1.5 ml siliconized polypropylene microtubes (Sigma-Aldrich, T4816) and stored at -80°C until required. The absorbance maximum of free hemoglobin (at 414 nm) was measured to detect free hemoglobin in serum samples, and serum with absorbance values < 0.2 was used for further analysis [8]. MiRNAs were isolated from 200 *μ*l serum using the miRCURY RNA Isolation Kit-Biofluids (300112; Exiqon), according to the manufacturer's instructions. The quantity of miRNA was measured using the MyQubit microRNA Assay. The small RNA fraction was detected using the RNA 6000 NanoKit (Agilent) and a Bioanalyzer 2100. Samples that passed the quality check were stored at -80°C until required.

Peripheral blood was collected using the Tempus RNA Isolation Kit (Thermo Fisher Scientific), and total RNA was isolated according to the manufacturer's instructions. RNA quality and quantity were analyzed using a NanoDrop spectrophotometer, and samples with A260/A280 ratios of 1.8–2.1 were assessed further using an Agilent 2100 Bioanalyzer.

Preparation of an NGS library, sequencing, and data analyses were conducted as described previously [13]. Unmapped bam files were converted into fastq files using a bamToFastq script from bedtools [14]. Read mapping to the human genome (hg19), quantification of known miRNAs (according to miRBase release 18) [15], and prediction of novel miRNAs were performed using miRDeep2 [16].

### 2.4. Statistical Analyses

PLS-DA and sPLS-DA modeling was performed with mixOmics package, according to the guidelines present in a tutorial on http://mixomics.org/case-studies/splsda-srbct/. The optimal number of components was chosen based on differences in the mean error rate between components as measured by centroid distances. The final model error rate was computed using 5-fold cross-validation, repeated 100 times. The analysis was performed on samples before treatment and healthy controls. Differential expression of miRNAs was analyzed using edgeR [17]. Areas under the receiver operating characteristic curve (AUC-ROC) values were calculated using R in the pROC package [18]. Functional analysis was conducted using mirPath version 3 [19], with the gene intersection (for at least two miRNAs) and conservative statistic options.

## 3. Results

### 3.1. Sequencing Results

Profiles of c-miRNAs (cohort no. 1) and wb-miRNAs (cohort no. 2) were analyzed by deep small RNA sequencing. The serum pretherapeutic samples were obtained from 20 HL patients and 21 DLBCL patients, and the posttherapeutic samples, obtained at the end of therapy, were also collected from 18 HL and in 17 DLBCL patients of the cohort no. 1. The whole blood pretherapeutic samples were available from 21 HL patients and 17 DLBCL patients, while the posttherapeutic samples, from 8 HL and 9 DLBCL patients of the cohort no. 2. The control group for each cohort comprised 30 healthy adults. On average, 386602 and 1834673 reads (mapped to miRBase) were obtained per library, representing 49% and 86% of total reads for c-miRNA and wb-miRNA, respectively. Altogether, 1284 and 1270 mature c-miRNAs and wb-miRNAs, respectively, were detected, of which 254 and 290, respectively, generated ten reads on average.

Due to the small number of the paired samples in cohort no. 2, all further comparisons were related to control samples.

### 3.2. Diagnostic Potential of c-miRNA Profiling

**Figure 1:**
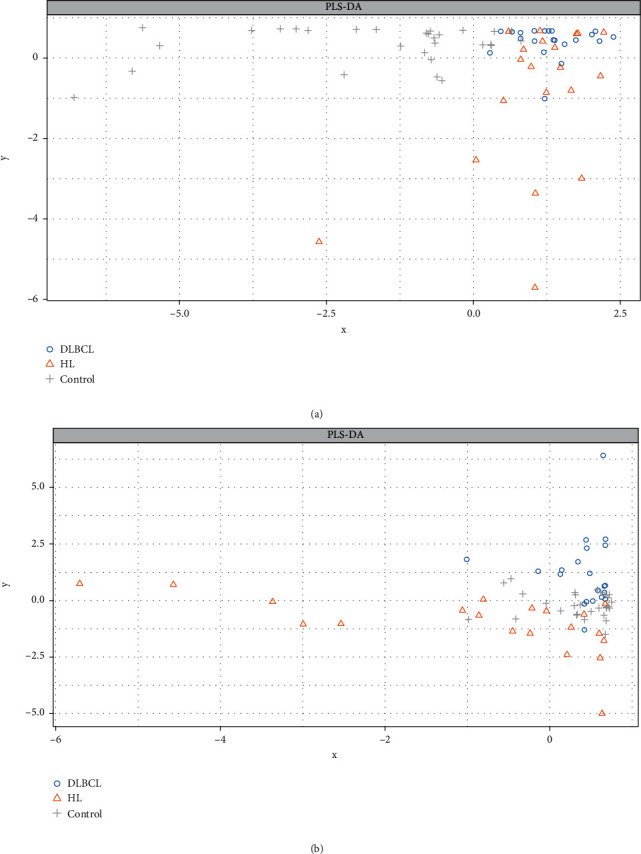
Individual sample plots of sPLS-DA modeling results for c-miRNAs, representing the first two (a) and second and third components (b).

The optimal sPLS-DA model consisted of 3 components, with 15, 5, and 15 variables, respectively. Sample plots indicate that healthy controls are separated from the other groups on the first component, while DLBCLs and HLs before treatment are separated mainly on the third component (Figure 1). Overall, 24 miRNAs contributed to differentiation between outcomes: 11 had maximum median values for controls, 10 for HLs, and only 4 for DLBCL (Supplementary Figure 1).

### 3.3. Differential Expression of c-miRNAs

**Figure 2:**
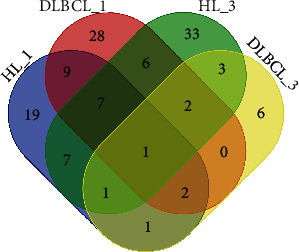
Venn diagram showing lymphoma patients and controls with respect to expression of c-miRNA, where 1 indicates samples before treatment and 3 indicates samples after treatment.

Pairwise comparisons identified 125 c-miRNAs (adjusted *P* value < 0.05) showing significant dysregulation between the 30 healthy controls and patients; of these, 47 and 55 differentiated controls from pretherapeutic HL and DLBCL patients, respectively. Log-fold changes ranged from -2.7 to 6.15 and from −2.65 to 6.12, respectively. Sixty and sixteen c-miRNAs differentiated controls from posttherapeutic HL and DLBCL patients, respectively, with log-fold changes ranging from −3.3 to 6.54 and −3.37 to 5.95, respectively (Figure 2; Supplementary Table S1).

One dysregulated c-miRNA (hsa-miR-101-3p) was common to all four pairwise comparisons (Figure 2), whereas seven (hsa-miR-320c, hsa-miR-3676-5p, hsa-miR-320b, hsa-miR-320d, hsa-miR-7-1-3p, hsa-miR-4454, and hsa-miR-532-3p) and no c-miRNAs were common to pre- and posttherapeutic HL and DLBCL patients, respectively. Nine (hsa-miR-4466, hsa-miR-4492, hsa-miR-9-3p, hsa-miR-4772-5p, hsa-miR-151b, hsa-miR-126-3p, hsa-miR-129-5p, hsa-miR-151a-5p, and hsa-miR-4792) and three (hsa-miR-483-5p, hsa-miR-431-3p, and hsa-miR-483-3p) c-miRNAs were common to both pre- and posttherapeutic lymphoma patients, respectively. In addition, 19, 33, 28, and six miRNAs were unique to pre- and posttherapeutic HL and pre- and posttherapeutic DLBCL patient comparisons, respectively (Supplementary Table S1).

### 3.4. Diagnostic Potential of wb-miRNA Profiling

**Figure 3:**
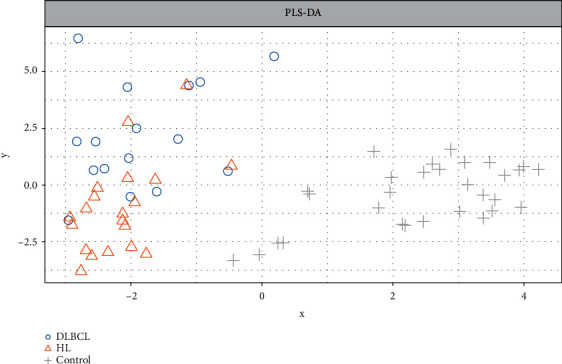
Individual sample plots of sPLS-DA modeling results for wb-miRNAs, representing first two components.

The optimal sPLS-DA model consisted of 2 components, with 15 variables each. Sample plots indicate that healthy controls are separated from the other groups on the first component, while DLBCLs and HLs before treatment are separated mainly on second component (Figure 3). Overall, 29 miRNAs contributed to differentiation between outcomes: 7 had maximum median values for controls, 6 for HLs and 16 for DLBCLs (Supplementary Figure 2).

### 3.5. Diagnostic Potential and Functional Analysis of wb-miRNAs

**Figure 4:**
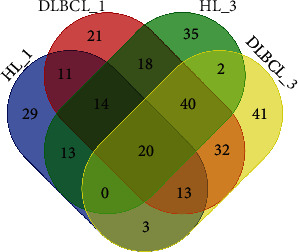
Venn diagram showing lymphoma patients and controls with respect to expression of wb-miRNA, where 1 indicates samples before treatment and 3 indicates samples after treatment.

Pairwise comparisons identified 292 wb-miRNAs (adjusted *P* value < 0.05) showing significant dysregulation between the 30 controls and patients; of these, 103 and 169 differentiated controls from pretherapeutic HL and DLBCL patients, respectively. Log-fold changes ranged from −2.88 to 3.57 and −3.32 to 3.83, respectively. In addition, 142 and 151 wb-miRNAs differentiated controls from posttherapeutic HL and DLBCL patients, respectively, with log-fold changes ranging from −4.12 to 3.96 and −3.74 to 5.35, respectively (Figure 4; Supplementary Table S2).

Of the dysregulated wb-miRNAs, 20 (hsa-miR-378i, hsa-miR-503, hsa-miR-378c, hsa-miR-4454, hsa-miR-99a-5p, hsa-miR-4690-3p, hsa-miR-378d, hsa-let-7i-3p, hsa-miR-199b-5p, hsa-miR-20a-3p, hsa-miR-34a-5p, hsa-miR-92a-3p, hsa-miR-421, hsa-miR-652-5p, hsa-miR-1537, hsa-miR-31-3p, hsa-miR-378f, hsa-miR-362-5p, hsa-miR-124-3p, and hsa-miR-4785) were common to all four pairwise comparisons (Figure 4). Thirteen and thirty-two dysregulated wb-miRNAs were common to pre- and posttherapeutic HL and DLBCL patients, respectively, and 11 and two miRNAs were common to both pre- and posttherapeutic lymphoma patients, respectively. Another 29 and 35 wb-miRNAs were unique to pre- and posttherapeutic HL patients, respectively, and 21 and 41 were unique to pre- and posttherapeutic DLBCL patients, respectively (Supplementary Table S2). Functional analysis revealed that 20 common dysregulated miRNAs play roles in regulating 35 signaling pathways, including the p53, MAPK, and ErbB pathways (Supplementary Table S3). Fourteen miRNAs (hsa-miR-31-3p, hsa-miR-124-3p, hsa-miR-421, hsa-miR-378f, hsa-miR-199b-5p, hsa-miR-362-5p, hsa-miR-378d, hsa-miR-20a-3p, hsa-miR-378i, hsa-miR-92a-3p, hsa-miR-34a-5p, hsa-miR-99a-5p, hsa-miR-378c, and hsa-miR-652-5p) played a role in regulating all 35 pathways.

### 3.6. Comparison of miRNA PLS-DA Models

Overall, wb-miRNA sPLS-DA had much lower error rate than c-miRNA sPLS-DA (0.27 versus 0.52). As far as individual outcomes are concerned, HLs and DLBCLs had similar error rates in c-miRNA sPLS-DA while in wb-miRNA sPLS-DA, DLBCLs present much higher error rate. The control group has the lowest error rates in both models (Table 2).

### 3.7. Comparison of miRNA Profiles

To note, the profiles of dysregulated circulating and whole blood miRNAs differed significantly. Of the 145 and 186 c-miRNAs and/or wb-miRNAs dysregulated in pre- and posttherapeutic HL patients, respectively, only five and 16, respectively, were common to both sets of miRNAs. A similar comparison among DLBCL patients identified 213 and 164 c-miRNAs and/or wb-miRNAs that were dysregulated in pre- and posttherapeutic patients, respectively, of which 11 and three, respectively, were common to both miRNA sets. Furthermore, when the diagnostic potential of dysregulated miRNAs was assessed using ROC curve and AUC analyses, only one c-miRNA (hsa-miR-34a-5p) showed high (excellent) discriminatory power (AUC-ROC values = 0.924) for differentiating healthy controls from posttherapeutic HL patients; by contrast, 61 wb-miRNAs exhibited high discriminatory power, with AUC-ROC values ranging from 0.9 to 1.0 (Table 3).

## 4. Discussion

While pathological diagnosis is the gold standard in the field of clinical oncology, precise diagnosis in hematooncology requires testing for karyotype and genetic variants. However, of the thousands of potential cancer molecular biomarkers reported every year, few add value to conventional oncological diagnostic methods [20]. Selection of new biomarkers depends strictly on the choice of starting material in a discovery step.

Here, we performed deep sequencing of small RNAs and compared expression profiles in serum and whole blood samples from patients with HL or DLBCL. Of 145 and 186 c-miRNAs/wb-miRNAs dysregulated in pre-/posttherapeutic HL patients, respectively, 49 and 60, respectively, were dysregulated in serum and 103 and 142, respectively, were dysregulated in whole blood. Of 213 and 164 c-miRNAs/wb-miRNAs dysregulated in pre-/posttherapeutic DLBCL patients, respectively, 55 and 16, respectively, were dysregulated in serum samples and 169 and 151, respectively, were dysregulated in whole blood. Only single elements were common to both lists of dysregulated miRNAs. Moreover, the profiles of dysregulated circulating and whole blood miRNAs differed not only in terms of the number of differentiated miRNAs but also in terms of their diagnostic potential. Only one c-miRNA showed high discriminatory power (AUC-ROC value ≥ 0.9) for differentiating healthy controls from posttherapeutic HL patients, whereas 61 wb-miRNAs differentiated controls from HL and/or DLBCL patients. PLS-DA profiles had lower error rates for wb-miRNAs and a smaller number of components was needed to differentiate between conditions.

MiRNAs are abundant, noncoding RNA molecules of 18–25 nucleotides that regulate cellular development, proliferation, differentiation, and apoptosis, thereby contributing to numerous human diseases, including cancer [5]. Lawrie et al. [21] provided the first description of altered levels of miRNAs (including miR-155, miR-210, and miR-21) in serum from DLBCL patients, which subsequently were reported widely [22–30]. However, while at least two studies identified only seven circulating miRNAs (miR-15a, miR-21, miR-29c, miR-34a, miR-145, miR-155, and miR-210) as deregulated significantly in DLBCL patients, five of them yielded conflicting results [2]. Two studies identified miR-145 as consistently downregulated in DLBCL patients [26, 27], and six of eight studies that examined miR-21 found it to be upregulated [21, 23, 25, 26, 28, 31]. Of the circulating miRNAs identified to date that show clinical or prognostic implications for those with HL [9, 32–36] (summarized recently in [6]), most were common to two or more of the six studies of miRNA signatures. However, due to relatively low number of recruited patients, we were unable to assess the predictive value of circulating miRNAs in the treatment response.

Blood comes into contact with cells, tissues, and organs within an organism and constitutes a major component of the immune system; therefore, changes of gene expression by white blood cells (WBCs) are associated with a wide range of pathological conditions. In fact, blood is a surrogate for traditional tissue specimens used for clinical diagnosis; as such, analysis of WBC expression profiles is a noninvasive method that supports other traditional methods [7]. Repeated measurement of expression profiles in whole blood from healthy subjects can generate reliable and consistent data over several months [37]; indeed, specific profiles are associated with a wide range of diseases, including autoimmune and inflammatory diseases, infectious disorders, psychiatric, cardiovascular, neurological, and neoplastic diseases [38, 39]. Links between environmental factors and disease have been identified. For example, blood transcriptome analysis identified associations between socioeconomic status and chronic inflammation [40] and species- and strain-specific discrimination of infectious diseases [41]. Other studies stratified patients according to disease progression before and after the onset of type 1 diabetes [42], predicted the responsiveness of systemic lupus erythematosus and rheumatoid arthritis to anti-IFN therapy [43], and identified alterations in WBC gene expression profiles in those with inflammatory bowel disease [8].

When expression of miR-9, miR-20a, miR-21, miR-26a, and miR-155 was analyzed in whole blood samples from pretherapy and posttherapy HL patients, all differed significantly after ABVD treatment, indicating their potential as biomarkers of treatment responses [44]. Our study revealed 292 whole blood microRNAs, compared to 125 circulating miRNAs, dysregulated between lymphoma patients and healthy controls. Furthermore, dysregulated whole blood miRNAs showed also much higher diagnostic potentials, when assessed by the AUC analyses, as compared to those resulting from the dysregulated circulating microRNAs.

However, as c-miRNA and wb-RNA profiling involved two different patient cohorts, our study shows significant limitations due to the heterogeneity of the analyzed samples and the lack of paired samples. It is also not clear if alterations in the whole blood miRNAs may reflect profiles of lymphoma cells or the changes in the kind and quantity of circulating blood cells or both the options.

## 5. Conclusions

Although the nature of the whole blood miRNA changes is unclear, the results presented herein suggest that the whole blood miRNAs may be a comparable or even a better source of lymphoma candidate biomarkers than miRNAs circulating in serum.

## Figures and Tables

**Table 1:** Patient characteristics.

	COHORT 1		COHORT 2	
	DLBCL, *n* = 21	HL, *n* = 20	DLBCL, *n* = 23	HL, *n* = 20
Male	14 (67%)	5 (25%)	2 (17%)	5 (24%)
Age, median (range)	61 (24–82)	33 (19–68)	65 (39–82)	31 (23–68)
CS III/IV	13 (62%)	7 (35%)	15 (88%)	7 (33%)
B symptoms	10 (48%)	7 (35%)	8 (47%)	11 (52%)
Extranodal involvement	15 (71%)	5 (25%)	9 (52%)	4 (19%)
Bulky disease (more than 5 cm)	7 (33%)	9 (45%)	5 (25%)	10 (47%)

DLBCL: diffuse B cell large lymphoma; HL: Hodgkin lymphoma; CS: clinical status according to the Ann Arbor staging system. Data are expressed as number (percentage) or as the median (range). 18 HL/17 DLBCL and eight HL/nine DLBCL patients, respectively, after treatment.

**Table 2:** Error rates for wb-miRNA and c-miRNA sPLS-DA models.

	wb-miRNA	c-miRNA
Overall	0.27	0.52
DLBCL	0.48	0.60
HL	0.29	0.62
Control	0.14	0.40

**Table 3:** miRNAs with high diagnostic potential (AUC > 0.9) in comparison with healthy controls.

Name	Total number	Element
DLBCL-1 DLBCL-3 HL-1 HL-3	3	hsa-miR-92a-3p, hsa-miR-378c, hsa-miR-362-5p
DLBCL-1 DLBCL-3 HL-3	6	hsa-let-7a-5p, hsa-miR-98, hsa-let-7f-5p, hsa-miR-106a-5p, hsa-miR-210, hsa-miR-26a-5p
DLBCL-1 HL-3	1	hsa-let-7d-5p
DLBCL-3 HL-3	5	hsa-miR-34a-5p, hsa-miR-185-5p, hsa-miR-421, hsa-miR-4785, hsa-miR-652-5p
DLBCL-1 DLBCL-3	15	hsa-miR-181b-5p, hsa-miR-192-5p, hsa-miR-20b-5p, hsa-miR-502-3p, hsa-miR-29b-3p, hsa-miR-29c-3p, hsa-miR-378d, hsa-miR-363-3p, hsa-miR-500a-3p, hsa-miR-502-5p, hsa-let-7c, hsa-miR-103a-3p, hsa-miR-4454, hsa-miR-29c-5p, hsa-miR-140-3p
HL-3	7	hsa-miR-21-5p, hsa-miR-190a, hsa-let-7b-5p, hsa-let-7e-5p, hsa-miR-574-5p, hsa-miR-342-5p, hsa-miR-629-5p
DLBCL-1	1	hsa-miR-1537
DLBCL-3	23	hsa-miR-3130-5p, hsa-miR-330-5p, hsa-miR-503, hsa-miR-296-5p, hsa-miR-3909, hsa-miR-18b-5p, hsa-miR-18a-5p, hsa-miR-378i, hsa-miR-194-5p, hsa-miR-126-3p, hsa-miR-598, hsa-miR-425-5p, hsa-miR-222-3p, hsa-miR-589-3p, hsa-miR-17-3p, hsa-miR-660-5p, hsa-miR-454-3p, hsa-miR-1273c, hsa-miR-33a-3p, hsa-miR-501-5p, hsa-miR-151a-3p, hsa-miR-378a-3p, hsa-miR-625-5p

Name: name of comparison with healthy controls, where 1 indicates before treatment and 3 indicates after treatment.

## Data Availability

The raw data from this study are available as uBAM files in the European Nucleotide Archive under accession number PRJEB32681.

## Supplementary Materials

Supplementary Materials Supplementary Table S1: the results of differential expression test performed with edgeR for c-miRNAs, where logFC stands for fold-change logarithm, LR stands for likelihood ratio test statistic, PValue stands for *P* value in likelihood ratio test, QValue stands for *P* value after FDR correction, and AUC stands for area under the ROC curve. Supplementary Table S2: the results of differential expression test performed with edgeR for wb-miRNAs, where logFC stands for fold-change logarithm, LR stands for likelihood ratio test statistic, PValue stands for *P* value in likelihood ratio test, QValue stands for *P* value after FDR correction, and AUC stands for area under the ROC curve. Supplementary Table S3: pathways influenced by 20 common dysregulated miRNAs in whole blood samples. Supplementary Figure 1: contribution of variables to each sPLS-DA component in c-miRNAs. The bars are coloured according to outcome for which variable has a maximal median value. Supplementary Figure 2: contribution of variables to each sPLS-DA component in wb-miRNAs. The bars are coloured according to outcome for which variable has a maximal median value.
