# Supplementary material for: Peripheral Blood Cells from Patients with Hodgkin's and Diffuse Large B Cell Lymphomas May Be a Better Source of Candidate Diagnostic miRNAs Than Circulating miRNAs
Source: Biomed Res Int. 2021 Feb 4;2021:3212878. doi: 10.1155/2021/3212878 (PMC7880712; doi:10.1155/2021/3212878)

Supplementary Table S1. The results of differential expression test performed with edgeR for c-miRNAs, where logFC - fold-change logarithm, LR - likelihood ratio statistic, Pvalue - p-value in likelihood ratio test, Qvalue - p-value after FDR correction, AUC - area under ROC curve.

	logFC	LR	PValue	QValue	AUC	Comparison
hsa-miR-206	-2.71197	11.11952	0.000854	0.02464	0.675	HL_1
hsa-miR-203	-1.6835	11.32809	0.000763	0.02464	0.728333	HL_1
hsa-miR-495	-1.55229	11.74804	0.000609	0.021883	0.69	HL_1
hsa-miR-340-3p	-1.26816	10.14823	0.001444	0.033024	0.734167	HL_1
hsa-miR-150-5p	-1.17665	13.27714	0.000269	0.012566	0.776667	HL_1
hsa-miR-126-3p	-0.9524	13.97908	0.000185	0.012537	0.743333	HL_1
hsa-miR-151a-5p	-0.86972	16.33327	5.31E-05	0.007738	0.776667	HL_1
hsa-miR-101-3p	-0.85054	11.62034	0.000652	0.022561	0.66	HL_1
hsa-miR-151b	-0.84688	16.46462	4.96E-05	0.007738	0.775	HL_1
hsa-miR-7-1-3p	1.000564	10.7222	0.001059	0.026268	0.688333	HL_1
hsa-let-7d-3p	1.0217	13.94475	0.000188	0.012537	0.708333	HL_1
hsa-miR-629-5p	1.066126	13.69942	0.000215	0.012566	0.75	HL_1
hsa-miR-320b	1.079829	11.85805	0.000574	0.021392	0.725	HL_1
hsa-miR-532-3p	1.089236	10.72746	0.001056	0.026268	0.713333	HL_1
hsa-miR-582-5p	1.105949	14.19475	0.000165	0.012537	0.7975	HL_1
hsa-miR-338-5p	1.126176	13.83679	0.000199	0.012537	0.721667	HL_1
hsa-miR-378a-5p	1.189778	11.56291	0.000673	0.022561	0.746667	HL_1
hsa-miR-501-3p	1.197115	15.43435	8.54E-05	0.008682	0.751667	HL_1
hsa-miR-345-5p	1.200155	12.1888	0.000481	0.018695	0.770833	HL_1
hsa-miR-551a	1.281416	10.83112	0.000998	0.026268	0.713333	HL_1
hsa-miR-205-5p	1.294768	9.580304	0.001967	0.043012	0.49	HL_1
hsa-miR-193a-5p	1.349899	16.30778	5.38E-05	0.007738	0.691667	HL_1
hsa-miR-320c	1.409628	12.66379	0.000373	0.016306	0.74	HL_1
hsa-miR-320d	1.428306	9.420637	0.002146	0.045924	0.7175	HL_1
hsa-miR-642a-5p	1.434538	11.06077	0.000882	0.02464	0.633333	HL_1
hsa-miR-4454	1.437169	10.70134	0.001071	0.026268	0.614167	HL_1
hsa-miR-4772-5p	1.478009	9.87358	0.001677	0.037483	0.78	HL_1
hsa-miR-877-5p	1.569449	13.54485	0.000233	0.012566	0.5875	HL_1
hsa-miR-365a-3p	1.673313	11.19401	0.000821	0.02464	0.725	HL_1
hsa-miR-365b-3p	1.673319	11.19683	0.000819	0.02464	0.725	HL_1
hsa-miR-188-5p	1.678609	12.24039	0.000468	0.018695	0.7375	HL_1
hsa-miR-4466	2.087595	10.32799	0.00131	0.031384	0.690833	HL_1
hsa-miR-3940-5p	2.105771	10.18007	0.00142	0.033024	0.6	HL_1
hsa-miR-29b-1-5p	2.148588	13.25512	0.000272	0.012566	0.620833	HL_1
hsa-miR-27a-5p	2.157147	14.11593	0.000172	0.012537	0.658333	HL_1
hsa-miR-9-3p	2.237655	18.45611	1.74E-05	0.00583	0.73	HL_1
hsa-miR-34c-5p	2.289491	12.17947	0.000483	0.018695	0.641667	HL_1
hsa-miR-34c-3p	2.60852	11.09013	0.000868	0.02464	0.6	HL_1
hsa-miR-618	2.6433	18.98077	1.32E-05	0.00583	0.773333	HL_1
hsa-miR-4792	2.752415	10.94553	0.000938	0.025511	0.665833	HL_1
hsa-miR-4772-3p	3.046634	13.3089	0.000264	0.012566	0.583333	HL_1
hsa-miR-1285-5p	3.119654	17.42083	3E-05	0.007533	0.615833	HL_1
hsa-miR-211-5p	3.130707	13.9061	0.000192	0.012537	0.6625	HL_1
hsa-miR-129-2-3p	3.4885	13.23473	0.000275	0.012566	0.57	HL_1
hsa-miR-4492	3.991991	15.85725	6.83E-05	0.008589	0.626667	HL_1
hsa-miR-129-5p	6.061504	32.19346	1.4E-08	1.4E-05	0.7	HL_1
hsa-miR-3676-5p	6.15019	15.41496	8.63E-05	0.008682	0.560833	HL_1
hsa-miR-369-3p	-2.65491	27.88598	1.29E-07	0.00013	0.831746	DLBCL_1
hsa-miR-154-3p	-2.08184	13.33731	0.00026	0.010553	0.715873	DLBCL_1
hsa-miR-411-3p	-1.87805	9.835236	0.001712	0.037739	0.748413	DLBCL_1
hsa-miR-376a-5p	-1.82038	10.08605	0.001494	0.03523	0.81746	DLBCL_1
hsa-miR-495	-1.72944	15.64053	7.66E-05	0.004996	0.72381	DLBCL_1
hsa-miR-190b	-1.72056	10.73685	0.00105	0.025974	0.795238	DLBCL_1
hsa-miR-136-3p	-1.70448	12.11842	0.000499	0.015413	0.810317	DLBCL_1
hsa-miR-24-2-5p	-1.63048	15.95539	6.49E-05	0.004996	0.738095	DLBCL_1
hsa-miR-487b	-1.57673	11.81205	0.000588	0.017049	0.69127	DLBCL_1
hsa-miR-382-3p	-1.56595	9.56379	0.001985	0.041923	0.696825	DLBCL_1
hsa-miR-1185-1-3p	-1.5451	9.430629	0.002134	0.044159	0.694444	DLBCL_1
hsa-miR-379-5p	-1.46997	9.202045	0.002417	0.047531	0.753968	DLBCL_1
hsa-miR-1	-1.11766	9.117857	0.002531	0.047531	0.706349	DLBCL_1
hsa-miR-190a	-1.09093	14.52384	0.000138	0.006683	0.771429	DLBCL_1
hsa-miR-101-3p	-1.00875	15.51975	8.16E-05	0.004996	0.719048	DLBCL_1
hsa-miR-301a-3p	-0.96548	12.87554	0.000333	0.011356	0.774603	DLBCL_1
hsa-miR-126-3p	-0.94526	14.84689	0.000117	0.006223	0.750794	DLBCL_1
hsa-miR-144-5p	-0.88972	17.61916	2.7E-05	0.003909	0.844444	DLBCL_1
hsa-miR-151a-5p	-0.81594	13.90784	0.000192	0.008781	0.784127	DLBCL_1

hsa-miR-151b	-0.7903	13.83888	0.000199	0.008781	0.784127	DLBCL_1
hsa-miR-501-3p	0.906744	11.63682	0.000647	0.018107	0.807937	DLBCL_1
hsa-miR-629-5p	0.925223	10.40532	0.001257	0.030336	0.744444	DLBCL_1
hsa-miR-486-3p	1.042922	9.594772	0.001951	0.041923	0.612698	DLBCL_1
hsa-miR-210	1.050823	11.24269	0.000799	0.020784	0.585714	DLBCL_1
hsa-miR-193a-5p	1.123015	13.00558	0.000311	0.011247	0.734921	DLBCL_1
hsa-miR-345-5p	1.194738	12.85828	0.000336	0.011356	0.707143	DLBCL_1
hsa-miR-378a-5p	1.220004	11.46904	0.000708	0.018883	0.669841	DLBCL_1
hsa-miR-4772-5p	1.39867	9.141816	0.002498	0.047531	0.731746	DLBCL_1
hsa-miR-188-5p	1.465009	10.02363	0.001545	0.035616	0.681746	DLBCL_1
hsa-miR-193b-3p	1.48585	13.11549	0.000293	0.011247	0.698413	DLBCL_1
hsa-miR-34a-5p	1.520942	16.89212	3.96E-05	0.004996	0.74127	DLBCL_1
hsa-miR-1537	1.57856	12.5411	0.000398	0.013022	0.595238	DLBCL_1
hsa-miR-629-3p	1.832047	9.931775	0.001624	0.036605	0.553968	DLBCL_1
hsa-miR-365a-3p	1.843087	15.51973	8.16E-05	0.004996	0.742857	DLBCL_1
hsa-miR-365b-3p	1.843097	15.52303	8.15E-05	0.004996	0.742857	DLBCL_1
hsa-miR-3960	1.872197	9.185297	0.00244	0.047531	0.647619	DLBCL_1
hsa-miR-500a-5p	1.89817	11.93477	0.000551	0.016432	0.6	DLBCL_1
hsa-miR-500b	1.949633	11.59665	0.000661	0.018107	0.571429	DLBCL_1
hsa-miR-3192	1.958722	10.80862	0.00101	0.025611	0.595238	DLBCL_1
hsa-miR-1228-5p	2.081105	15.66145	7.58E-05	0.004996	0.721429	DLBCL_1
hsa-miR-4792	2.170316	13.02126	0.000308	0.011247	0.65873	DLBCL_1
hsa-miR-3150b-5p	2.22676	13.4573	0.000244	0.010311	0.619048	DLBCL_1
hsa-miR-1246	2.240011	15.1625	9.86E-05	0.005557	0.787302	DLBCL_1
hsa-miR-9-3p	2.331778	15.75205	7.22E-05	0.004996	0.631746	DLBCL_1
hsa-miR-4707-3p	2.408312	9.335077	0.002248	0.045591	0.680952	DLBCL_1
hsa-miR-34a-3p	2.416727	14.68409	0.000127	0.006445	0.652381	DLBCL_1
hsa-miR-4466	2.519845	12.10967	0.000502	0.015413	0.698413	DLBCL_1
hsa-miR-1273f	2.964669	19.27486	1.13E-05	0.003626	0.696825	DLBCL_1
hsa-miR-1290	3.199961	23.4702	1.27E-06	0.000643	0.68254	DLBCL_1
hsa-miR-4492	3.322191	15.55279	8.02E-05	0.004996	0.622222	DLBCL_1
hsa-miR-4488	3.591049	15.47156	8.38E-05	0.004996	0.649206	DLBCL_1
hsa-miR-129-5p	3.849677	9.056657	0.002617	0.048255	0.590476	DLBCL_1
hsa-miR-3689e	5.121043	18.06908	2.13E-05	0.003626	0.595238	DLBCL_1
hsa-miR-3689b-5p	5.121145	18.05596	2.15E-05	0.003626	0.595238	DLBCL_1
hsa-miR-3689a-5p	5.12115	18.05532	2.15E-05	0.003626	0.595238	DLBCL_1
hsa-miR-1185-2-3p	-3.29402	11.46257	0.00071	0.019666	0.3	HL_3
hsa-miR-369-5p	-2.90643	17.4448	2.96E-05	0.001698	0.844444	HL_3
hsa-miR-431-3p	-2.75474	11.05397	0.000885	0.022046	0.773148	HL_3
hsa-miR-382-3p	-2.15211	13.02927	0.000307	0.009671	0.798148	HL_3
hsa-miR-495	-1.99427	19.29716	1.12E-05	0.000797	0.753704	HL_3
hsa-miR-369-3p	-1.90464	14.81745	0.000118	0.004373	0.738889	HL_3
hsa-miR-487a	-1.66835	9.475067	0.002083	0.040717	0.747222	HL_3
hsa-miR-431-5p	-1.57233	10.46589	0.001216	0.026538	0.787037	HL_3
hsa-miR-150-5p	-1.47001	15.90597	6.66E-05	0.003017	0.82963	HL_3
hsa-miR-487b	-1.45578	8.996701	0.002705	0.047856	0.686111	HL_3
hsa-miR-323a-3p	-1.44525	8.916244	0.002826	0.047856	0.748148	HL_3
hsa-miR-494	-1.29908	10.4235	0.001244	0.026538	0.714815	HL_3
hsa-miR-376c	-1.09813	11.26427	0.00079	0.020955	0.716667	HL_3
hsa-miR-342-3p	-1.01322	13.00661	0.00031	0.009671	0.792593	HL_3
hsa-miR-101-3p	-0.88953	11.24426	0.000799	0.020955	0.677778	HL_3
hsa-miR-301a-3p	-0.87394	8.995989	0.002706	0.047856	0.775926	HL_3
hsa-miR-660-5p	0.661315	9.607722	0.001938	0.038636	0.788889	HL_3
hsa-miR-500a-3p	0.830911	11.0168	0.000903	0.022046	0.757407	HL_3
hsa-miR-532-3p	0.966186	9.056379	0.002618	0.047856	0.714815	HL_3
hsa-miR-99a-5p	1.014468	8.881988	0.00288	0.047856	0.611111	HL_3
hsa-miR-324-3p	1.023743	11.0092	0.000907	0.022046	0.742593	HL_3
hsa-miR-501-3p	1.026316	12.24918	0.000465	0.013649	0.792593	HL_3
hsa-miR-30a-5p	1.044317	15.55641	8.01E-05	0.003434	0.709259	HL_3
hsa-miR-7-1-3p	1.088989	10.57667	0.001145	0.026025	0.716667	HL_3
hsa-miR-100-5p	1.12333	13.2983	0.000266	0.008828	0.590741	HL_3
hsa-miR-1307-3p	1.260383	13.63977	0.000221	0.007613	0.635185	HL_3
hsa-miR-483-3p	1.262122	15.12154	0.000101	0.003866	0.77037	HL_3
hsa-miR-186-5p	1.289897	19.86341	8.32E-06	0.000668	0.709259	HL_3
hsa-miR-378i	1.30301	12.5898	0.000388	0.011718	0.646296	HL_3
hsa-miR-339-5p	1.330011	16.62875	4.55E-05	0.002266	0.590741	HL_3
hsa-miR-1537	1.410118	9.045928	0.002633	0.047856	0.639815	HL_3
hsa-miR-629-5p	1.429505	19.77539	8.71E-06	0.000668	0.775926	HL_3
hsa-miR-345-5p	1.477316	17.45207	2.95E-05	0.001698	0.783333	HL_3
hsa-miR-769-3p	1.498893	10.15035	0.001443	0.029967	0.611111	HL_3

hsa-miR-378a-5p	1.502374	17.11609	3.52E-05	0.001845	0.774074	HL_3
hsa-miR-627	1.624606	15.199	9.68E-05	0.003859	0.685185	HL_3
hsa-miR-4454	1.666269	13.93497	0.000189	0.006738	0.575	HL_3
hsa-miR-378a-3p	1.688206	23.29698	1.39E-06	0.000126	0.67037	HL_3
hsa-miR-378c	1.688325	15.49618	8.27E-05	0.003434	0.612963	HL_3
hsa-miR-1228-5p	1.732162	10.63329	0.001111	0.026025	0.702778	HL_3
hsa-miR-34a-5p	1.84237	34.169	5.05E-09	7.2E-07	0.924074	HL_3
hsa-miR-188-5p	2.06311	15.92204	6.6E-05	0.003017	0.675	HL_3
hsa-miR-3191-3p	2.09742	10.57131	0.001149	0.026025	0.583333	HL_3
hsa-miR-769-5p	2.150797	28.74723	8.25E-08	9.14E-06	0.601852	HL_3
hsa-miR-642a-3p	2.158599	9.079261	0.002585	0.047856	0.521296	HL_3
hsa-miR-195-3p	2.161739	8.943389	0.002785	0.047856	0.598148	HL_3
hsa-miR-148a-5p	2.253241	12.02444	0.000525	0.014957	0.653704	HL_3
hsa-miR-940	2.328139	10.41343	0.001251	0.026538	0.601852	HL_3
hsa-miR-483-5p	2.350307	33.80633	6.09E-09	7.59E-07	0.798148	HL_3
hsa-miR-4999-5p	2.379443	9.619989	0.001925	0.038636	0.62037	HL_3
hsa-miR-193a-5p	2.691278	41.38045	1.25E-10	3.12E-08	0.766667	HL_3
hsa-miR-320d	2.710559	25.79827	3.79E-07	3.78E-05	0.775	HL_3
hsa-miR-5100	2.719006	17.37653	3.07E-05	0.001698	0.517593	HL_3
hsa-miR-1307-5p	2.785175	35.63515	2.38E-09	3.95E-07	0.744444	HL_3
hsa-miR-320c	2.834617	38.70656	4.93E-10	9.82E-08	0.853704	HL_3
hsa-miR-320a	2.956527	48.47688	3.34E-12	1.67E-09	0.722222	HL_3
hsa-miR-320b	3.176639	49.96566	1.56E-12	1.56E-09	0.785185	HL_3
hsa-miR-147b	3.232239	8.896792	0.002857	0.047856	0.52037	HL_3
hsa-miR-34a-3p	3.379765	42.01063	9.08E-11	3.02E-08	0.773148	HL_3
hsa-miR-3676-5p	6.541329	18.70307	1.53E-05	0.001015	0.598148	HL_3
hsa-miR-431-3p	-3.37331	13.0264	0.000307	0.020127	0.8	DLBCL_3
hsa-miR-3613-3p	-3.02223	15.6851	7.48E-05	0.006685	0.782353	DLBCL_3
hsa-miR-150-5p	-1.26897	11.7768	0.0006	0.036846	0.776471	DLBCL_3
hsa-miR-101-3p	-0.96509	13.04515	0.000304	0.020127	0.694118	DLBCL_3
hsa-miR-450b-5p	1.374899	16.86266	4.02E-05	0.004389	0.673529	DLBCL_3
hsa-miR-483-5p	1.440466	13.66643	0.000218	0.016509	0.684314	DLBCL_3
hsa-miR-483-3p	1.441225	16.02788	6.24E-05	0.006136	0.760784	DLBCL_3
hsa-miR-885-5p	1.724873	19.40676	1.06E-05	0.001483	0.813725	DLBCL_3
hsa-miR-34a-5p	1.969824	31.07651	2.48E-08	1.22E-05	0.868627	DLBCL_3
hsa-miR-365a-3p	2.293767	22.91043	1.7E-06	0.000278	0.882353	DLBCL_3
hsa-miR-365b-3p	2.293773	22.90918	1.7E-06	0.000278	0.882353	DLBCL_3
hsa-miR-211-5p	3.362912	17.82317	2.42E-05	0.002979	0.665686	DLBCL_3
hsa-miR-34a-3p	3.566101	38.10346	6.71E-10	6.6E-07	0.786275	DLBCL_3
hsa-miR-517b-3p	3.652127	28.01728	1.2E-07	2.95E-05	0.708824	DLBCL_3
hsa-miR-517a-3p	3.652155	28.03553	1.19E-07	2.95E-05	0.708824	DLBCL_3
hsa-miR-3151	5.9486	14.34724	0.000152	0.012452	0.619608	DLBCL_3

Supplementary Table S2. The results of differential expression test performed with edgeR for wb-miRNAs, where logFC - fold-change logarithm, LR - likelihood ratio test statistic, Pvalue - p-value in likelihood ratio test, Qvalue - p-value after FDR correction, AUC - area under ROC curve.

	logFC	LR	PValue	QValue	AUC	Comparison
hsa-miR-1234	-2.88	18.05	2.15E-05	6.59E-04	0.28	HL_1
hsa-miR-1	-2.79	10.81	1.01E-03	1.20E-02	0.58	HL_1
hsa-miR-3591-3p	-2.64	15.27	9.31E-05	2.01E-03	0.32	HL_1
hsa-miR-4722-5p	-2.63	13.60	2.27E-04	4.20E-03	0.32	HL_1
hsa-miR-1296	-2.39	20.36	6.40E-06	3.01E-04	0.74	HL_1
hsa-miR-31-3p	-2.34	10.50	1.19E-03	1.32E-02	0.33	HL_1
hsa-miR-4738-3p	-2.27	10.34	1.31E-03	1.38E-02	0.35	HL_1
hsa-miR-3187-3p	-2.26	9.47	2.09E-03	2.03E-02	0.36	HL_1
hsa-miR-92a-3p	-2.22	12.20	4.78E-04	7.45E-03	0.95	HL_1
hsa-miR-3064-5p	-2.17	12.83	3.41E-04	5.91E-03	0.73	HL_1
hsa-miR-4655-5p	-2.15	10.35	1.29E-03	1.38E-02	0.33	HL_1
hsa-miR-197-5p	-2.12	15.29	9.21E-05	2.01E-03	0.73	HL_1
hsa-miR-4722-3p	-2.07	8.97	2.75E-03	2.57E-02	0.37	HL_1
hsa-miR-4750	-2.04	11.36	7.52E-04	1.09E-02	0.76	HL_1
hsa-miR-146b-3p	-2.04	12.30	4.52E-04	7.36E-03	0.74	HL_1
hsa-miR-3196	-2.03	7.65	5.68E-03	4.53E-02	0.34	HL_1
hsa-miR-874	-2.03	7.47	6.26E-03	4.85E-02	0.40	HL_1
hsa-miR-3682-3p	-1.94	9.59	1.95E-03	1.95E-02	0.71	HL_1
hsa-miR-6741-3p	-1.87	10.33	1.31E-03	1.38E-02	0.65	HL_1
hsa-miR-548l	-1.87	17.34	3.13E-05	7.57E-04	0.75	HL_1
hsa-miR-3177-3p	-1.69	19.40	1.06E-05	4.21E-04	0.80	HL_1
hsa-miR-4659b-3p	-1.58	8.53	3.49E-03	3.16E-02	0.70	HL_1
hsa-miR-4685-3p	-1.58	28.27	1.05E-07	1.66E-05	0.81	HL_1
hsa-let-7i-3p	-1.55	19.10	1.24E-05	4.30E-04	0.76	HL_1
hsa-miR-3173-5p	-1.55	14.96	1.10E-04	2.26E-03	0.72	HL_1
hsa-miR-4742-3p	-1.53	13.38	2.54E-04	4.59E-03	0.76	HL_1
hsa-miR-1304-5p	-1.51	20.80	5.10E-06	2.90E-04	0.79	HL_1
hsa-miR-23a-5p	-1.46	9.14	2.50E-03	2.37E-02	0.64	HL_1
hsa-miR-200b-3p	-1.46	8.49	3.58E-03	3.20E-02	0.69	HL_1
hsa-miR-1303	-1.44	19.85	8.38E-06	3.51E-04	0.83	HL_1
hsa-miR-1306-3p	-1.44	20.25	6.81E-06	3.01E-04	0.79	HL_1
hsa-miR-4448	-1.39	10.77	1.03E-03	1.21E-02	0.73	HL_1
hsa-miR-122-5p	-1.33	8.06	4.51E-03	3.90E-02	0.68	HL_1
hsa-miR-4732-3p	-1.32	19.31	1.11E-05	4.21E-04	0.77	HL_1
hsa-miR-1294	-1.32	24.02	9.53E-07	7.59E-05	0.86	HL_1
hsa-miR-939	-1.29	12.17	4.86E-04	7.45E-03	0.82	HL_1
hsa-miR-6741-5p	-1.28	12.53	4.01E-04	6.66E-03	0.75	HL_1
hsa-miR-636	-1.23	17.48	2.91E-05	7.24E-04	0.83	HL_1
hsa-miR-328	-1.21	12.85	3.38E-04	5.91E-03	0.72	HL_1
hsa-miR-190b	-1.21	10.90	9.59E-04	1.19E-02	0.70	HL_1
hsa-miR-21-3p	-1.15	10.14	1.45E-03	1.50E-02	0.67	HL_1
hsa-miR-3605-5p	-1.14	8.71	3.16E-03	2.93E-02	0.76	HL_1
hsa-miR-548b-5p	-1.08	7.63	5.74E-03	4.53E-02	0.72	HL_1
hsa-miR-6793-3p	-1.06	10.44	1.23E-03	1.35E-02	0.73	HL_1
hsa-miR-5010-5p	-1.05	8.64	3.30E-03	3.02E-02	0.67	HL_1

hsa-miR-1976	-0.98	7.93	4.87E-03	4.04E-02	0.69	HL_1
hsa-miR-1270	-0.94	11.00	9.10E-04	1.19E-02	0.72	HL_1
hsa-miR-193a-5p	-0.86	7.71	5.49E-03	4.46E-02	0.75	HL_1
hsa-miR-532-5p	-0.82	11.24	8.02E-04	1.11E-02	0.75	HL_1
hsa-miR-1255b-5p	-0.78	7.98	4.72E-03	3.96E-02	0.73	HL_1
hsa-miR-454-5p	0.56	8.03	4.59E-03	3.90E-02	0.81	HL_1
hsa-miR-548ap-3p	0.68	8.20	4.19E-03	3.67E-02	0.75	HL_1
hsa-miR-335-5p	0.77	10.05	1.53E-03	1.56E-02	0.76	HL_1
hsa-miR-589-3p	0.79	7.50	6.17E-03	4.82E-02	0.74	HL_1
hsa-miR-7-1-3p	0.84	12.55	3.95E-04	6.66E-03	0.79	HL_1
hsa-miR-19b-1-5p	0.84	10.94	9.39E-04	1.19E-02	0.63	HL_1
hsa-miR-421	0.85	17.99	2.22E-05	6.59E-04	0.86	HL_1
hsa-miR-378i	0.86	9.51	2.04E-03	2.01E-02	0.68	HL_1
hsa-miR-33a-3p	0.87	7.86	5.06E-03	4.16E-02	0.74	HL_1
hsa-miR-151a-3p	0.87	11.63	6.49E-04	9.75E-03	0.77	HL_1
hsa-miR-28-3p	0.87	10.63	1.11E-03	1.27E-02	0.77	HL_1
hsa-let-7f-1-3p	0.91	11.23	8.04E-04	1.11E-02	0.74	HL_1
hsa-miR-99a-5p	0.94	14.89	1.14E-04	2.27E-03	0.83	HL_1
hsa-miR-503	0.95	17.72	2.56E-05	6.80E-04	0.82	HL_1
hsa-miR-143-3p	0.95	10.75	1.04E-03	1.21E-02	0.65	HL_1
hsa-miR-3909	0.98	11.18	8.25E-04	1.12E-02	0.69	HL_1
hsa-miR-641	0.99	10.91	9.56E-04	1.19E-02	0.66	HL_1
hsa-let-7b-3p	1.04	11.22	8.07E-04	1.11E-02	0.66	HL_1
hsa-miR-150-3p	1.06	9.28	2.32E-03	2.23E-02	0.66	HL_1
hsa-miR-625-5p	1.06	17.97	2.25E-05	6.59E-04	0.78	HL_1
hsa-miR-576-3p	1.09	13.94	1.88E-04	3.66E-03	0.77	HL_1
hsa-miR-551b-3p	1.11	9.62	1.92E-03	1.94E-02	0.62	HL_1
hsa-miR-502-5p	1.13	13.73	2.11E-04	4.01E-03	0.80	HL_1
hsa-miR-378d	1.20	16.82	4.10E-05	9.62E-04	0.76	HL_1
hsa-miR-22-5p	1.25	27.50	1.57E-07	1.66E-05	0.84	HL_1
hsa-miR-376c	1.26	17.91	2.31E-05	6.59E-04	0.77	HL_1
hsa-miR-338-3p	1.30	18.35	1.84E-05	6.11E-04	0.75	HL_1
hsa-miR-551a	1.41	7.64	5.71E-03	4.53E-02	0.65	HL_1
hsa-miR-652-5p	1.43	27.87	1.30E-07	1.66E-05	0.86	HL_1
hsa-miR-296-5p	1.45	17.65	2.65E-05	6.81E-04	0.78	HL_1
hsa-miR-376a-3p	1.47	17.83	2.42E-05	6.65E-04	0.81	HL_1
hsa-miR-494	1.48	19.10	1.24E-05	4.30E-04	0.80	HL_1
hsa-miR-20a-3p	1.50	23.22	1.44E-06	1.05E-04	0.83	HL_1
hsa-miR-136-5p	1.51	8.04	4.58E-03	3.90E-02	0.73	HL_1
hsa-miR-4690-3p	1.55	12.17	4.85E-04	7.45E-03	0.68	HL_1
hsa-miR-199b-5p	1.58	30.28	3.75E-08	7.47E-06	0.79	HL_1
hsa-miR-181d	1.61	8.20	4.19E-03	3.67E-02	0.66	HL_1
hsa-miR-378c	1.65	56.91	4.57E-14	3.64E-11	0.96	HL_1
hsa-miR-4454	1.66	27.39	1.66E-07	1.66E-05	0.83	HL_1
hsa-miR-362-5p	1.68	31.59	1.91E-08	5.06E-06	0.92	HL_1
hsa-miR-542-3p	1.72	22.09	2.60E-06	1.59E-04	0.85	HL_1
hsa-miR-2355-5p	1.72	10.96	9.29E-04	1.19E-02	0.71	HL_1
hsa-miR-3074-3p	1.79	10.89	9.67E-04	1.19E-02	0.68	HL_1
hsa-miR-492	1.86	10.56	1.16E-03	1.30E-02	0.63	HL_1
hsa-miR-3683	1.87	11.54	6.82E-04	1.01E-02	0.70	HL_1
hsa-miR-378f	1.95	20.52	5.90E-06	3.01E-04	0.73	HL_1

hsa-miR-1537	2.09	22.22	2.43E-06	1.59E-04	0.79	HL_1
hsa-miR-34c-5p	2.20	10.83	1.00E-03	1.20E-02	0.63	HL_1
hsa-miR-1226-5p	2.23	16.66	4.46E-05	1.02E-03	0.65	HL_1
hsa-miR-4785	2.41	20.33	6.50E-06	3.01E-04	0.76	HL_1
hsa-miR-34a-3p	2.78	14.95	1.10E-04	2.26E-03	0.63	HL_1
hsa-miR-34a-5p	3.33	43.29	4.72E-11	1.88E-08	0.73	HL_1
hsa-miR-124-3p	3.57	25.05	5.59E-07	4.95E-05	0.75	HL_1
hsa-miR-1	-3.32	10.88	9.72E-04	7.02E-03	0.52	DLBCL_1
hsa-miR-4695-3p	-2.52	10.13	1.46E-03	9.78E-03	0.34	DLBCL_1
hsa-miR-31-3p	-2.48	11.32	7.67E-04	5.64E-03	0.34	DLBCL_1
hsa-let-7c	-2.39	50.77	1.04E-12	1.00E-10	0.96	DLBCL_1
hsa-miR-4443	-2.37	9.14	2.50E-03	1.54E-02	0.37	DLBCL_1
hsa-miR-6741-3p	-2.35	14.46	1.43E-04	1.33E-03	0.66	DLBCL_1
hsa-miR-1228-3p	-2.30	12.45	4.17E-04	3.39E-03	0.33	DLBCL_1
hsa-miR-3591-3p	-2.21	10.12	1.47E-03	9.78E-03	0.36	DLBCL_1
hsa-miR-874	-2.17	7.98	4.73E-03	2.54E-02	0.40	DLBCL_1
hsa-miR-92a-3p	-2.11	68.97	1.00E-16	1.54E-14	0.95	DLBCL_1
hsa-miR-935	-1.95	7.80	5.24E-03	2.75E-02	0.38	DLBCL_1
hsa-miR-1234	-1.93	8.94	2.80E-03	1.62E-02	0.34	DLBCL_1
hsa-miR-149-5p	-1.93	7.86	5.06E-03	2.68E-02	0.39	DLBCL_1
hsa-miR-335-3p	-1.82	9.00	2.69E-03	1.60E-02	0.67	DLBCL_1
hsa-miR-1275	-1.80	8.52	3.52E-03	1.94E-02	0.35	DLBCL_1
hsa-miR-193b-3p	-1.75	9.04	2.64E-03	1.59E-02	0.69	DLBCL_1
hsa-miR-4738-3p	-1.71	7.48	6.25E-03	3.16E-02	0.45	DLBCL_1
hsa-miR-150-5p	-1.58	19.74	8.88E-06	1.16E-04	0.84	DLBCL_1
hsa-miR-3064-5p	-1.51	7.67	5.60E-03	2.90E-02	0.61	DLBCL_1
hsa-miR-939	-1.44	15.94	6.55E-05	6.57E-04	0.83	DLBCL_1
hsa-let-7i-3p	-1.42	16.45	5.00E-05	5.22E-04	0.75	DLBCL_1
hsa-miR-1180	-1.41	22.21	2.44E-06	3.70E-05	0.85	DLBCL_1
hsa-miR-1270	-1.34	19.83	8.46E-06	1.13E-04	0.86	DLBCL_1
hsa-miR-1304-5p	-1.33	15.91	6.66E-05	6.59E-04	0.74	DLBCL_1
hsa-miR-92b-3p	-1.32	27.83	1.32E-07	3.10E-06	0.86	DLBCL_1
hsa-miR-3150b-3p	-1.32	7.85	5.08E-03	2.68E-02	0.69	DLBCL_1
hsa-let-7f-5p	-1.28	78.85	6.70E-19	2.59E-16	0.98	DLBCL_1
hsa-miR-4742-3p	-1.26	11.67	6.37E-04	4.77E-03	0.74	DLBCL_1
hsa-miR-23a-5p	-1.21	7.44	6.37E-03	3.20E-02	0.60	DLBCL_1
hsa-miR-574-5p	-1.21	18.76	1.48E-05	1.84E-04	0.82	DLBCL_1
hsa-miR-342-5p	-1.20	23.06	1.57E-06	2.58E-05	0.87	DLBCL_1
hsa-let-7b-5p	-1.16	24.31	8.21E-07	1.51E-05	0.84	DLBCL_1
hsa-miR-629-5p	-1.10	25.62	4.15E-07	8.66E-06	0.85	DLBCL_1
hsa-let-7a-5p	-1.08	69.60	7.27E-17	1.40E-14	0.96	DLBCL_1
hsa-miR-4448	-1.07	6.92	8.55E-03	4.05E-02	0.69	DLBCL_1
hsa-miR-1301	-1.04	13.67	2.18E-04	1.91E-03	0.80	DLBCL_1
hsa-miR-98	-0.97	33.37	7.62E-09	2.80E-07	0.95	DLBCL_1
hsa-miR-342-3p	-0.94	13.96	1.87E-04	1.70E-03	0.79	DLBCL_1
hsa-miR-6741-5p	-0.94	6.68	9.74E-03	4.53E-02	0.69	DLBCL_1
hsa-miR-1287	-0.94	12.11	5.01E-04	3.91E-03	0.76	DLBCL_1
hsa-miR-4732-3p	-0.93	9.10	2.55E-03	1.55E-02	0.70	DLBCL_1
hsa-let-7e-5p	-0.93	20.30	6.62E-06	8.97E-05	0.86	DLBCL_1
hsa-miR-636	-0.93	9.03	2.66E-03	1.59E-02	0.78	DLBCL_1
hsa-miR-190b	-0.92	6.61	1.01E-02	4.66E-02	0.69	DLBCL_1

hsa-miR-4685-3p	-0.89	8.08	4.48E-03	2.42E-02	0.72	DLBCL_1
hsa-miR-4433b-5p	-0.88	7.15	7.49E-03	3.61E-02	0.69	DLBCL_1
hsa-miR-1303	-0.87	7.74	5.39E-03	2.81E-02	0.75	DLBCL_1
hsa-miR-423-5p	-0.80	9.67	1.87E-03	1.19E-02	0.73	DLBCL_1
hsa-let-7d-5p	-0.77	24.68	6.77E-07	1.31E-05	0.92	DLBCL_1
hsa-miR-1294	-0.75	8.35	3.85E-03	2.11E-02	0.75	DLBCL_1
hsa-miR-2110	-0.75	8.75	3.10E-03	1.77E-02	0.75	DLBCL_1
hsa-let-7g-5p	-0.75	25.23	5.08E-07	1.03E-05	0.87	DLBCL_1
hsa-miR-15b-5p	-0.68	10.42	1.24E-03	8.65E-03	0.76	DLBCL_1
hsa-miR-584-5p	-0.65	10.50	1.19E-03	8.44E-03	0.74	DLBCL_1
hsa-let-7i-5p	-0.54	6.56	1.05E-02	4.78E-02	0.72	DLBCL_1
hsa-miR-451a	0.40	13.46	2.44E-04	2.09E-03	0.80	DLBCL_1
hsa-miR-454-3p	0.43	7.27	7.00E-03	3.42E-02	0.76	DLBCL_1
hsa-miR-107	0.51	18.66	1.57E-05	1.89E-04	0.84	DLBCL_1
hsa-miR-23b-3p	0.53	7.48	6.25E-03	3.16E-02	0.71	DLBCL_1
hsa-miR-101-3p	0.63	10.64	1.11E-03	7.91E-03	0.72	DLBCL_1
hsa-miR-424-5p	0.64	7.04	7.95E-03	3.81E-02	0.67	DLBCL_1
hsa-miR-185-5p	0.64	12.83	3.41E-04	2.83E-03	0.81	DLBCL_1
hsa-miR-27a-3p	0.64	8.72	3.15E-03	1.79E-02	0.77	DLBCL_1
hsa-miR-140-5p	0.64	7.29	6.92E-03	3.40E-02	0.73	DLBCL_1
hsa-miR-361-3p	0.65	8.26	4.05E-03	2.20E-02	0.74	DLBCL_1
hsa-miR-126-3p	0.65	11.13	8.49E-04	6.18E-03	0.78	DLBCL_1
hsa-miR-27b-3p	0.66	8.69	3.19E-03	1.80E-02	0.76	DLBCL_1
hsa-miR-19a-3p	0.67	14.70	1.26E-04	1.20E-03	0.78	DLBCL_1
hsa-miR-29a-3p	0.69	12.75	3.55E-04	2.92E-03	0.79	DLBCL_1
hsa-miR-93-5p	0.69	10.01	1.56E-03	1.01E-02	0.68	DLBCL_1
hsa-miR-28-5p	0.69	10.00	1.57E-03	1.01E-02	0.76	DLBCL_1
hsa-miR-374b-5p	0.70	16.54	4.77E-05	5.05E-04	0.80	DLBCL_1
hsa-miR-17-3p	0.71	18.21	1.98E-05	2.28E-04	0.87	DLBCL_1
hsa-miR-17-5p	0.72	17.30	3.19E-05	3.62E-04	0.82	DLBCL_1
hsa-miR-18b-5p	0.73	20.99	4.62E-06	6.73E-05	0.87	DLBCL_1
hsa-miR-21-5p	0.74	22.28	2.36E-06	3.64E-05	0.84	DLBCL_1
hsa-miR-148b-5p	0.74	10.31	1.33E-03	8.98E-03	0.66	DLBCL_1
hsa-miR-324-5p	0.74	10.33	1.31E-03	8.92E-03	0.79	DLBCL_1
hsa-miR-660-3p	0.74	10.46	1.22E-03	8.55E-03	0.80	DLBCL_1
hsa-miR-301a-3p	0.75	13.36	2.57E-04	2.18E-03	0.77	DLBCL_1
hsa-miR-18a-5p	0.75	23.77	1.09E-06	1.90E-05	0.88	DLBCL_1
hsa-miR-548ap-3p	0.75	11.87	5.71E-04	4.37E-03	0.85	DLBCL_1
hsa-miR-660-5p	0.75	19.49	1.01E-05	1.28E-04	0.85	DLBCL_1
hsa-miR-335-5p	0.76	8.99	2.71E-03	1.60E-02	0.75	DLBCL_1
hsa-miR-769-5p	0.76	7.15	7.48E-03	3.61E-02	0.76	DLBCL_1
hsa-miR-598	0.77	7.34	6.73E-03	3.33E-02	0.73	DLBCL_1
hsa-miR-363-3p	0.79	23.75	1.10E-06	1.90E-05	0.91	DLBCL_1
hsa-miR-19b-3p	0.82	27.29	1.75E-07	3.97E-06	0.85	DLBCL_1
hsa-miR-106a-3p	0.83	8.55	3.46E-03	1.92E-02	0.71	DLBCL_1
hsa-miR-7-1-3p	0.85	13.05	3.03E-04	2.54E-03	0.85	DLBCL_1
hsa-miR-103a-3p	0.85	55.07	1.16E-13	1.28E-11	0.96	DLBCL_1
hsa-miR-222-3p	0.87	20.50	5.95E-06	8.20E-05	0.85	DLBCL_1
hsa-miR-23a-3p	0.88	18.73	1.51E-05	1.85E-04	0.81	DLBCL_1
hsa-miR-625-5p	0.88	13.93	1.89E-04	1.70E-03	0.75	DLBCL_1
hsa-miR-33a-3p	0.90	10.01	1.56E-03	1.01E-02	0.86	DLBCL_1

hsa-miR-150-3p	0.90	7.53	6.06E-03	3.10E-02	0.71	DLBCL_1
hsa-miR-425-5p	0.91	27.84	1.32E-07	3.10E-06	0.81	DLBCL_1
hsa-miR-616-5p	0.91	7.35	6.72E-03	3.33E-02	0.76	DLBCL_1
hsa-miR-185-3p	0.92	8.97	2.74E-03	1.60E-02	0.71	DLBCL_1
hsa-miR-641	0.93	9.59	1.96E-03	1.23E-02	0.70	DLBCL_1
hsa-miR-330-5p	0.93	6.70	9.66E-03	4.52E-02	0.69	DLBCL_1
hsa-miR-151a-3p	0.93	13.87	1.96E-04	1.74E-03	0.85	DLBCL_1
hsa-miR-186-5p	0.93	20.82	5.04E-06	7.07E-05	0.85	DLBCL_1
hsa-miR-501-5p	0.93	12.40	4.30E-04	3.46E-03	0.80	DLBCL_1
hsa-miR-421	0.94	19.53	9.89E-06	1.27E-04	0.89	DLBCL_1
hsa-miR-503	0.95	17.15	3.46E-05	3.81E-04	0.84	DLBCL_1
hsa-miR-16-2-3p	0.95	15.53	8.14E-05	7.95E-04	0.75	DLBCL_1
hsa-miR-590-5p	0.95	21.77	3.08E-06	4.57E-05	0.83	DLBCL_1
hsa-miR-20b-5p	0.95	30.34	3.62E-08	1.08E-06	0.92	DLBCL_1
hsa-miR-106a-5p	0.96	31.18	2.36E-08	7.76E-07	0.92	DLBCL_1
hsa-miR-576-3p	0.97	9.66	1.88E-03	1.19E-02	0.81	DLBCL_1
hsa-miR-142-5p	0.97	24.35	8.02E-07	1.51E-05	0.85	DLBCL_1
hsa-miR-143-3p	0.99	14.57	1.35E-04	1.27E-03	0.80	DLBCL_1
hsa-miR-497-5p	1.03	10.38	1.27E-03	8.76E-03	0.77	DLBCL_1
hsa-miR-192-5p	1.06	43.93	3.41E-11	2.19E-09	0.91	DLBCL_1
hsa-miR-589-3p	1.07	11.43	7.22E-04	5.36E-03	0.76	DLBCL_1
hsa-miR-188-5p	1.08	18.53	1.67E-05	1.98E-04	0.86	DLBCL_1
hsa-miR-210	1.08	40.54	1.93E-10	1.06E-08	0.92	DLBCL_1
hsa-miR-326	1.08	16.09	6.05E-05	6.15E-04	0.77	DLBCL_1
hsa-miR-215	1.11	11.69	6.28E-04	4.75E-03	0.81	DLBCL_1
hsa-miR-500a-3p	1.12	36.49	1.54E-09	6.58E-08	0.93	DLBCL_1
hsa-miR-194-5p	1.12	28.27	1.06E-07	2.81E-06	0.84	DLBCL_1
hsa-miR-338-3p	1.13	16.54	4.77E-05	5.05E-04	0.77	DLBCL_1
hsa-miR-20a-3p	1.14	18.41	1.78E-05	2.08E-04	0.84	DLBCL_1
hsa-miR-30d-5p	1.14	25.92	3.56E-07	7.62E-06	0.81	DLBCL_1
hsa-miR-378a-3p	1.15	31.13	2.41E-08	7.76E-07	0.87	DLBCL_1
hsa-miR-542-3p	1.15	9.25	2.35E-03	1.47E-02	0.76	DLBCL_1
hsa-miR-339-5p	1.16	23.67	1.14E-06	1.92E-05	0.85	DLBCL_1
hsa-miR-502-3p	1.18	42.86	5.88E-11	3.49E-09	0.95	DLBCL_1
hsa-miR-181a-3p	1.26	12.30	4.53E-04	3.60E-03	0.77	DLBCL_1
hsa-miR-26a-5p	1.27	48.92	2.67E-12	2.06E-10	0.92	DLBCL_1
hsa-miR-4286	1.27	22.74	1.85E-06	2.92E-05	0.80	DLBCL_1
hsa-miR-378i	1.29	27.99	1.22E-07	3.10E-06	0.85	DLBCL_1
hsa-miR-181b-5p	1.30	37.84	7.69E-10	3.49E-08	0.90	DLBCL_1
hsa-miR-29c-5p	1.31	49.41	2.08E-12	1.79E-10	0.96	DLBCL_1
hsa-miR-99a-5p	1.37	24.95	5.88E-07	1.16E-05	0.88	DLBCL_1
hsa-miR-199b-5p	1.37	29.54	5.48E-08	1.57E-06	0.81	DLBCL_1
hsa-miR-551a	1.41	9.99	1.57E-03	1.01E-02	0.76	DLBCL_1
hsa-miR-652-5p	1.43	27.92	1.27E-07	3.10E-06	0.89	DLBCL_1
hsa-miR-3120-3p	1.43	7.62	5.78E-03	2.98E-02	0.75	DLBCL_1
hsa-miR-3909	1.44	28.43	9.74E-08	2.68E-06	0.84	DLBCL_1
hsa-miR-502-5p	1.44	30.34	3.63E-08	1.08E-06	0.97	DLBCL_1
hsa-miR-3117-3p	1.47	9.13	2.51E-03	1.54E-02	0.72	DLBCL_1
hsa-miR-140-3p	1.47	39.65	3.04E-10	1.56E-08	0.90	DLBCL_1
hsa-miR-1273c	1.49	17.10	3.55E-05	3.86E-04	0.85	DLBCL_1
hsa-miR-3687	1.49	8.76	3.09E-03	1.77E-02	0.70	DLBCL_1

hsa-miR-188-3p	1.55	15.39	8.73E-05	8.42E-04	0.75	DLBCL_1
hsa-miR-4690-3p	1.61	16.14	5.88E-05	6.05E-04	0.78	DLBCL_1
hsa-miR-3176	1.61	8.59	3.37E-03	1.89E-02	0.79	DLBCL_1
hsa-miR-548at-3p	1.63	6.63	1.00E-02	4.63E-02	0.70	DLBCL_1
hsa-miR-3922-5p	1.64	12.16	4.90E-04	3.86E-03	0.75	DLBCL_1
hsa-miR-4714-3p	1.70	20.88	4.89E-06	6.99E-05	0.73	DLBCL_1
hsa-miR-29b-3p	1.70	38.60	5.20E-10	2.51E-08	0.94	DLBCL_1
hsa-miR-30a-5p	1.73	13.65	2.20E-04	1.91E-03	0.71	DLBCL_1
hsa-miR-378f	1.74	17.21	3.34E-05	3.74E-04	0.80	DLBCL_1
hsa-miR-378d	1.76	45.86	1.27E-11	8.91E-10	0.91	DLBCL_1
hsa-miR-362-5p	1.80	33.93	5.72E-09	2.21E-07	0.94	DLBCL_1
hsa-miR-296-5p	1.84	26.43	2.73E-07	6.02E-06	0.86	DLBCL_1
hsa-miR-4772-3p	1.84	6.92	8.53E-03	4.05E-02	0.64	DLBCL_1
hsa-miR-548f	1.87	14.15	1.69E-04	1.55E-03	0.73	DLBCL_1
hsa-miR-1537	1.88	23.73	1.11E-06	1.90E-05	0.90	DLBCL_1
hsa-miR-490-3p	1.88	6.88	8.71E-03	4.10E-02	0.64	DLBCL_1
hsa-miR-4454	1.93	34.98	3.34E-09	1.36E-07	0.93	DLBCL_1
hsa-miR-378c	2.11	91.11	1.36E-21	1.05E-18	0.98	DLBCL_1
hsa-miR-34c-5p	2.31	11.90	5.61E-04	4.33E-03	0.70	DLBCL_1
hsa-miR-4785	2.72	32.90	9.72E-09	3.41E-07	0.89	DLBCL_1
hsa-miR-29c-3p	2.72	77.52	1.32E-18	3.39E-16	0.97	DLBCL_1
hsa-miR-34a-5p	3.39	58.31	2.24E-14	2.88E-12	0.87	DLBCL_1
hsa-miR-124-3p	3.83	22.74	1.85E-06	2.92E-05	0.78	DLBCL_1
hsa-miR-3180-5p	-4.12	8.61	3.34E-03	2.12E-02	0.37	HL_3
hsa-miR-197-5p	-3.32	16.44	5.01E-05	1.02E-03	0.76	HL_3
hsa-miR-4725-3p	-3.15	13.19	2.81E-04	3.61E-03	0.73	HL_3
hsa-miR-6741-3p	-3.14	12.45	4.18E-04	4.57E-03	0.70	HL_3
hsa-miR-31-3p	-3.12	12.40	4.29E-04	4.61E-03	0.32	HL_3
hsa-miR-3591-3p	-2.95	12.56	3.93E-04	4.36E-03	0.32	HL_3
hsa-miR-3196	-2.89	8.07	4.50E-03	2.76E-02	0.33	HL_3
hsa-miR-4792	-2.77	10.26	1.36E-03	1.15E-02	0.35	HL_3
hsa-miR-4446-3p	-2.67	20.41	6.25E-06	1.83E-04	0.82	HL_3
hsa-miR-3064-5p	-2.63	11.38	7.42E-04	7.15E-03	0.67	HL_3
hsa-miR-335-3p	-2.54	9.76	1.79E-03	1.38E-02	0.73	HL_3
hsa-miR-4443	-2.50	6.93	8.50E-03	4.57E-02	0.37	HL_3
hsa-miR-574-5p	-2.45	30.21	3.88E-08	2.37E-06	0.93	HL_3
hsa-miR-1228-3p	-2.44	10.11	1.47E-03	1.18E-02	0.33	HL_3
hsa-miR-204-5p	-2.42	7.68	5.58E-03	3.27E-02	0.38	HL_3
hsa-miR-3944-3p	-2.36	8.91	2.83E-03	1.90E-02	0.37	HL_3
hsa-miR-4448	-2.32	12.80	3.46E-04	4.08E-03	0.79	HL_3
hsa-miR-4750	-2.29	9.56	1.99E-03	1.50E-02	0.72	HL_3
hsa-miR-92a-3p	-2.29	37.52	9.05E-10	1.09E-07	0.95	HL_3
hsa-miR-3177-3p	-2.23	15.10	1.02E-04	1.73E-03	0.85	HL_3
hsa-miR-4687-5p	-2.09	8.87	2.90E-03	1.91E-02	0.69	HL_3
hsa-miR-939	-2.07	12.71	3.63E-04	4.22E-03	0.84	HL_3
hsa-miR-1976	-2.06	12.62	3.82E-04	4.30E-03	0.83	HL_3
hsa-miR-30b-3p	-2.05	10.14	1.45E-03	1.18E-02	0.76	HL_3
hsa-miR-432-5p	-2.03	8.83	2.96E-03	1.94E-02	0.68	HL_3
hsa-miR-4301	-2.03	6.79	9.16E-03	4.82E-02	0.38	HL_3
hsa-miR-433	-2.01	9.20	2.42E-03	1.72E-02	0.72	HL_3
hsa-miR-1224-5p	-1.91	12.26	4.63E-04	4.84E-03	0.82	HL_3

hsa-miR-328	-1.91	13.24	2.74E-04	3.58E-03	0.83	HL_3
hsa-miR-485-3p	-1.89	12.62	3.82E-04	4.30E-03	0.75	HL_3
hsa-let-7c	-1.89	14.59	1.34E-04	2.18E-03	0.85	HL_3
hsa-miR-6741-5p	-1.87	9.82	1.72E-03	1.34E-02	0.79	HL_3
hsa-miR-7854-3p	-1.86	9.48	2.07E-03	1.55E-02	0.68	HL_3
hsa-miR-1306-3p	-1.86	17.41	3.01E-05	6.29E-04	0.84	HL_3
hsa-miR-671-3p	-1.86	10.93	9.47E-04	8.66E-03	0.74	HL_3
hsa-miR-4659b-3p	-1.85	7.39	6.57E-03	3.66E-02	0.67	HL_3
hsa-miR-3150b-3p	-1.82	7.46	6.30E-03	3.60E-02	0.68	HL_3
hsa-miR-1280	-1.81	14.76	1.22E-04	2.03E-03	0.78	HL_3
hsa-let-7i-3p	-1.79	12.83	3.41E-04	4.08E-03	0.78	HL_3
hsa-miR-3605-5p	-1.76	12.11	5.02E-04	5.17E-03	0.78	HL_3
hsa-miR-4747-5p	-1.74	9.30	2.29E-03	1.64E-02	0.70	HL_3
hsa-miR-1270	-1.73	15.18	9.79E-05	1.73E-03	0.89	HL_3
hsa-let-7e-5p	-1.69	35.25	2.90E-09	2.65E-07	0.98	HL_3
hsa-miR-342-5p	-1.69	18.51	1.69E-05	3.87E-04	0.91	HL_3
hsa-let-7f-5p	-1.69	85.10	2.84E-20	6.93E-18	1.00	HL_3
hsa-miR-4433b-3p	-1.66	7.70	5.54E-03	3.27E-02	0.73	HL_3
hsa-miR-766-5p	-1.65	7.38	6.59E-03	3.66E-02	0.68	HL_3
hsa-miR-629-5p	-1.63	27.36	1.69E-07	8.81E-06	0.93	HL_3
hsa-let-7a-5p	-1.60	96.25	1.01E-22	3.71E-20	1.00	HL_3
hsa-let-7b-5p	-1.53	20.62	5.61E-06	1.71E-04	0.94	HL_3
hsa-miR-150-5p	-1.53	11.71	6.23E-04	6.33E-03	0.80	HL_3
hsa-miR-1294	-1.53	13.36	2.58E-04	3.43E-03	0.87	HL_3
hsa-miR-4742-3p	-1.50	9.02	2.68E-03	1.85E-02	0.72	HL_3
hsa-miR-92b-3p	-1.49	13.45	2.45E-04	3.32E-03	0.88	HL_3
hsa-miR-4685-3p	-1.45	9.36	2.21E-03	1.60E-02	0.81	HL_3
hsa-miR-4732-3p	-1.45	11.06	8.82E-04	8.39E-03	0.78	HL_3
hsa-miR-1303	-1.44	7.39	6.55E-03	3.66E-02	0.79	HL_3
hsa-miR-1304-5p	-1.43	10.99	9.18E-04	8.50E-03	0.74	HL_3
hsa-let-7d-3p	-1.43	13.45	2.45E-04	3.32E-03	0.84	HL_3
hsa-miR-98	-1.43	37.24	1.05E-09	1.09E-07	0.99	HL_3
hsa-miR-636	-1.42	11.48	7.04E-04	6.87E-03	0.83	HL_3
hsa-miR-4433b-5p	-1.38	10.04	1.53E-03	1.22E-02	0.81	HL_3
hsa-miR-1180	-1.38	9.44	2.13E-03	1.56E-02	0.85	HL_3
hsa-miR-1301	-1.35	10.19	1.41E-03	1.18E-02	0.83	HL_3
hsa-miR-664-5p	-1.34	7.57	5.92E-03	3.44E-02	0.70	HL_3
hsa-miR-493-5p	-1.34	6.74	9.42E-03	4.86E-02	0.66	HL_3
hsa-miR-423-5p	-1.34	13.06	3.02E-04	3.75E-03	0.85	HL_3
hsa-miR-548ap-5p	-1.33	8.06	4.52E-03	2.76E-02	0.76	HL_3
hsa-miR-1255b-5p	-1.32	12.27	4.60E-04	4.84E-03	0.85	HL_3
hsa-miR-363-5p	-1.32	6.98	8.25E-03	4.47E-02	0.73	HL_3
hsa-miR-193a-5p	-1.27	8.90	2.86E-03	1.90E-02	0.80	HL_3
hsa-miR-3940-3p	-1.27	7.92	4.89E-03	2.95E-02	0.78	HL_3
hsa-miR-197-3p	-1.25	10.59	1.13E-03	1.00E-02	0.82	HL_3
hsa-miR-2110	-1.25	14.06	1.77E-04	2.59E-03	0.92	HL_3
hsa-miR-1304-3p	-1.20	9.63	1.92E-03	1.46E-02	0.73	HL_3
hsa-miR-101-5p	-1.18	11.54	6.83E-04	6.75E-03	0.88	HL_3
hsa-miR-1285-3p	-1.16	7.03	8.02E-03	4.38E-02	0.77	HL_3
hsa-miR-324-3p	-1.01	6.80	9.14E-03	4.82E-02	0.77	HL_3
hsa-miR-26b-3p	-0.95	8.20	4.19E-03	2.62E-02	0.75	HL_3

hsa-miR-155-5p	-0.94	7.56	5.97E-03	3.44E-02	0.79	HL_3
hsa-miR-190a	-0.91	10.83	1.00E-03	9.03E-03	0.92	HL_3
hsa-let-7d-5p	-0.88	15.34	8.96E-05	1.65E-03	0.92	HL_3
hsa-let-7g-5p	-0.62	9.03	2.66E-03	1.85E-02	0.72	HL_3
hsa-miR-660-5p	0.57	6.75	9.40E-03	4.86E-02	0.85	HL_3
hsa-miR-454-3p	0.58	6.78	9.24E-03	4.83E-02	0.75	HL_3
hsa-miR-19b-3p	0.72	14.01	1.81E-04	2.60E-03	0.85	HL_3
hsa-miR-223-3p	0.73	7.34	6.73E-03	3.70E-02	0.63	HL_3
hsa-miR-126-3p	0.75	8.46	3.62E-03	2.29E-02	0.78	HL_3
hsa-miR-590-5p	0.76	8.97	2.75E-03	1.88E-02	0.82	HL_3
hsa-miR-191-5p	0.78	13.14	2.90E-04	3.66E-03	0.78	HL_3
hsa-miR-107	0.79	19.08	1.25E-05	3.05E-04	0.78	HL_3
hsa-miR-18a-5p	0.80	14.19	1.65E-04	2.46E-03	0.84	HL_3
hsa-miR-210	0.84	15.82	6.97E-05	1.34E-03	0.95	HL_3
hsa-miR-363-3p	0.85	11.60	6.60E-04	6.62E-03	0.74	HL_3
hsa-miR-186-5p	0.90	10.44	1.23E-03	1.08E-02	0.76	HL_3
hsa-miR-199b-5p	0.91	10.70	1.07E-03	9.56E-03	0.85	HL_3
hsa-miR-181b-5p	0.91	10.36	1.29E-03	1.11E-02	0.78	HL_3
hsa-miR-652-5p	0.96	8.68	3.21E-03	2.08E-02	0.91	HL_3
hsa-miR-21-5p	0.97	24.67	6.80E-07	2.62E-05	0.82	HL_3
hsa-miR-503	0.97	9.96	1.60E-03	1.26E-02	0.81	HL_3
hsa-miR-20a-3p	0.98	7.70	5.52E-03	3.27E-02	0.81	HL_3
hsa-miR-378a-3p	0.99	12.93	3.23E-04	3.94E-03	0.78	HL_3
hsa-miR-378i	0.99	10.18	1.42E-03	1.18E-02	0.83	HL_3
hsa-miR-324-5p	1.00	8.92	2.82E-03	1.90E-02	0.73	HL_3
hsa-miR-29c-5p	1.02	14.31	1.55E-04	2.39E-03	0.80	HL_3
hsa-miR-192-5p	1.03	21.86	2.94E-06	9.78E-05	0.82	HL_3
hsa-miR-29a-3p	1.04	15.10	1.02E-04	1.73E-03	0.73	HL_3
hsa-miR-106a-5p	1.04	25.13	5.35E-07	2.17E-05	0.91	HL_3
hsa-miR-99a-5p	1.08	9.44	2.12E-03	1.56E-02	0.79	HL_3
hsa-miR-20b-5p	1.09	20.28	6.69E-06	1.88E-04	0.87	HL_3
hsa-miR-17-5p	1.12	28.01	1.21E-07	6.81E-06	0.90	HL_3
hsa-miR-421	1.13	17.56	2.78E-05	5.99E-04	0.95	HL_3
hsa-miR-30d-5p	1.15	13.88	1.95E-04	2.75E-03	0.67	HL_3
hsa-miR-185-5p	1.15	25.18	5.23E-07	2.17E-05	0.90	HL_3
hsa-miR-22-5p	1.17	10.99	9.16E-04	8.50E-03	0.68	HL_3
hsa-miR-103a-3p	1.19	60.42	7.66E-15	1.12E-12	0.87	HL_3
hsa-miR-500a-3p	1.20	17.57	2.77E-05	5.99E-04	0.75	HL_3
hsa-miR-215	1.20	8.15	4.30E-03	2.67E-02	0.90	HL_3
hsa-miR-148b-5p	1.21	19.46	1.03E-05	2.59E-04	0.83	HL_3
hsa-miR-26a-5p	1.26	34.77	3.72E-09	3.02E-07	0.92	HL_3
hsa-miR-222-3p	1.28	16.10	6.00E-05	1.19E-03	0.62	HL_3
hsa-miR-1537	1.29	6.82	9.03E-03	4.82E-02	0.79	HL_3
hsa-miR-502-3p	1.33	24.54	7.30E-07	2.67E-05	0.78	HL_3
hsa-miR-194-5p	1.37	20.95	4.73E-06	1.50E-04	0.70	HL_3
hsa-miR-4714-3p	1.44	7.43	6.42E-03	3.64E-02	0.81	HL_3
hsa-miR-140-3p	1.48	22.07	2.63E-06	9.15E-05	0.82	HL_3
hsa-miR-362-5p	1.54	14.29	1.56E-04	2.39E-03	0.91	HL_3
hsa-miR-181c-3p	1.57	7.91	4.91E-03	2.95E-02	0.74	HL_3
hsa-miR-378f	1.58	9.11	2.55E-03	1.79E-02	0.82	HL_3
hsa-miR-29c-3p	1.59	14.42	1.46E-04	2.32E-03	0.80	HL_3

hsa-miR-93-5p	1.59	32.12	1.45E-08	9.65E-07	0.79	HL_3
hsa-miR-4454	1.63	15.34	8.99E-05	1.65E-03	0.78	HL_3
hsa-miR-378d	1.66	19.78	8.70E-06	2.28E-04	0.74	HL_3
hsa-miR-378c	1.68	33.90	5.80E-09	4.25E-07	0.90	HL_3
hsa-miR-4690-3p	1.73	10.14	1.45E-03	1.18E-02	0.82	HL_3
hsa-miR-425-5p	1.74	64.86	8.06E-16	1.47E-13	0.88	HL_3
hsa-miR-188-3p	1.84	8.64	3.30E-03	2.12E-02	0.82	HL_3
hsa-miR-1307-5p	2.39	26.85	2.20E-07	1.07E-05	0.72	HL_3
hsa-miR-4785	2.44	26.32	2.89E-07	1.32E-05	0.98	HL_3
hsa-miR-124-3p	3.77	18.69	1.54E-05	3.63E-04	0.84	HL_3
hsa-miR-34a-3p	3.78	19.82	8.51E-06	2.28E-04	0.75	HL_3
hsa-miR-34a-5p	3.96	96.89	7.34E-23	3.71E-20	0.92	HL_3
hsa-miR-1	-3.74	6.87	8.78E-03	4.55E-02	0.47	DLBCL_3
hsa-miR-200b-3p	-3.23	13.90	1.92E-04	1.88E-03	0.75	DLBCL_3
hsa-miR-31-3p	-2.82	7.64	5.71E-03	3.22E-02	0.91	DLBCL_3
hsa-let-7c	-2.46	26.60	2.50E-07	5.22E-06	0.94	DLBCL_3
hsa-miR-651	-2.14	7.85	5.09E-03	2.90E-02	0.75	DLBCL_3
hsa-miR-92a-3p	-1.70	27.66	1.45E-07	3.29E-06	0.90	DLBCL_3
hsa-miR-150-5p	-1.50	12.30	4.54E-04	3.73E-03	0.80	DLBCL_3
hsa-let-7i-3p	-1.42	8.31	3.93E-03	2.39E-02	0.74	DLBCL_3
hsa-let-7f-5p	-1.28	63.15	1.92E-15	3.71E-13	1.00	DLBCL_3
hsa-let-7a-5p	-0.95	43.59	4.05E-11	1.74E-09	0.97	DLBCL_3
hsa-miR-98	-0.89	14.01	1.82E-04	1.82E-03	0.95	DLBCL_3
hsa-miR-151b	0.61	7.49	6.20E-03	3.40E-02	0.84	DLBCL_3
hsa-miR-17-5p	0.62	8.34	3.87E-03	2.37E-02	0.85	DLBCL_3
hsa-miR-18b-5p	0.64	8.70	3.19E-03	1.99E-02	0.93	DLBCL_3
hsa-miR-624-5p	0.69	6.81	9.08E-03	4.68E-02	0.70	DLBCL_3
hsa-miR-151a-5p	0.70	9.46	2.09E-03	1.45E-02	0.86	DLBCL_3
hsa-miR-27a-3p	0.71	6.69	9.68E-03	4.96E-02	0.81	DLBCL_3
hsa-miR-454-3p	0.71	10.58	1.15E-03	8.60E-03	0.93	DLBCL_3
hsa-miR-374b-5p	0.74	12.69	3.68E-04	3.16E-03	0.86	DLBCL_3
hsa-miR-101-3p	0.76	11.30	7.75E-04	6.12E-03	0.82	DLBCL_3
hsa-miR-501-3p	0.77	9.99	1.57E-03	1.11E-02	0.76	DLBCL_3
hsa-miR-16-2-3p	0.77	7.97	4.76E-03	2.79E-02	0.81	DLBCL_3
hsa-miR-331-5p	0.80	10.00	1.57E-03	1.11E-02	0.82	DLBCL_3
hsa-miR-625-3p	0.81	8.14	4.32E-03	2.57E-02	0.80	DLBCL_3
hsa-miR-191-5p	0.82	17.51	2.86E-05	3.74E-04	0.75	DLBCL_3
hsa-miR-660-5p	0.82	12.87	3.33E-04	2.93E-03	0.91	DLBCL_3
hsa-miR-28-5p	0.82	8.68	3.22E-03	1.99E-02	0.79	DLBCL_3
hsa-miR-93-3p	0.82	7.56	5.98E-03	3.34E-02	0.76	DLBCL_3
hsa-miR-548aa	0.82	7.91	4.93E-03	2.83E-02	0.82	DLBCL_3
hsa-miR-548t-3p	0.82	7.90	4.94E-03	2.83E-02	0.82	DLBCL_3
hsa-miR-505-3p	0.84	9.11	2.55E-03	1.66E-02	0.83	DLBCL_3
hsa-miR-106a-5p	0.86	14.79	1.20E-04	1.29E-03	0.94	DLBCL_3
hsa-miR-335-5p	0.86	7.45	6.33E-03	3.45E-02	0.77	DLBCL_3
hsa-miR-142-5p	0.86	11.13	8.49E-04	6.56E-03	0.83	DLBCL_3
hsa-miR-18a-5p	0.87	16.19	5.73E-05	6.71E-04	0.95	DLBCL_3
hsa-miR-196b-5p	0.88	8.89	2.87E-03	1.85E-02	0.89	DLBCL_3
hsa-miR-107	0.89	23.43	1.30E-06	2.23E-05	0.88	DLBCL_3
hsa-miR-21-5p	0.90	17.16	3.44E-05	4.19E-04	0.86	DLBCL_3
hsa-miR-652-3p	0.91	9.67	1.87E-03	1.30E-02	0.79	DLBCL_3

hsa-miR-23a-3p	0.93	12.15	4.90E-04	3.99E-03	0.79	DLBCL_3
hsa-miR-301a-3p	0.93	11.80	5.93E-04	4.78E-03	0.81	DLBCL_3
hsa-miR-140-5p	0.94	11.30	7.75E-04	6.12E-03	0.86	DLBCL_3
hsa-miR-199b-5p	0.94	8.74	3.11E-03	1.96E-02	0.86	DLBCL_3
hsa-miR-4286	0.97	9.11	2.54E-03	1.66E-02	0.79	DLBCL_3
hsa-miR-500b	0.97	8.15	4.31E-03	2.57E-02	0.76	DLBCL_3
hsa-miR-27b-3p	0.99	13.18	2.83E-04	2.57E-03	0.87	DLBCL_3
hsa-miR-106a-3p	0.99	6.94	8.42E-03	4.45E-02	0.43	DLBCL_3
hsa-miR-195-5p	1.00	7.37	6.65E-03	3.57E-02	0.68	DLBCL_3
hsa-miR-23b-3p	1.00	14.30	1.56E-04	1.62E-03	0.76	DLBCL_3
hsa-miR-130b-5p	1.00	10.89	9.66E-04	7.40E-03	0.86	DLBCL_3
hsa-miR-500a-5p	1.01	8.74	3.11E-03	1.96E-02	0.78	DLBCL_3
hsa-miR-363-3p	1.03	19.57	9.67E-06	1.34E-04	0.99	DLBCL_3
hsa-miR-99b-5p	1.04	7.40	6.53E-03	3.53E-02	0.74	DLBCL_3
hsa-miR-1306-5p	1.05	9.15	2.49E-03	1.66E-02	0.77	DLBCL_3
hsa-miR-550a-3-5p	1.06	12.52	4.02E-04	3.41E-03	0.80	DLBCL_3
hsa-miR-29a-3p	1.07	15.87	6.79E-05	7.84E-04	0.83	DLBCL_3
hsa-miR-1307-3p	1.07	9.39	2.18E-03	1.49E-02	0.77	DLBCL_3
hsa-miR-3912	1.07	6.93	8.49E-03	4.45E-02	0.75	DLBCL_3
hsa-miR-590-5p	1.08	17.29	3.21E-05	4.14E-04	0.90	DLBCL_3
hsa-miR-361-3p	1.09	14.29	1.57E-04	1.62E-03	0.87	DLBCL_3
hsa-miR-183-3p	1.09	12.95	3.19E-04	2.84E-03	0.69	DLBCL_3
hsa-miR-17-3p	1.10	22.49	2.11E-06	3.34E-05	0.99	DLBCL_3
hsa-miR-550a-5p	1.10	13.83	2.00E-04	1.93E-03	0.80	DLBCL_3
hsa-miR-378a-5p	1.13	11.23	8.06E-04	6.29E-03	0.81	DLBCL_3
hsa-miR-93-5p	1.15	20.96	4.68E-06	6.96E-05	0.86	DLBCL_3
hsa-miR-185-5p	1.16	26.29	2.94E-07	5.68E-06	0.90	DLBCL_3
hsa-miR-150-3p	1.17	9.16	2.48E-03	1.66E-02	0.79	DLBCL_3
hsa-miR-126-3p	1.19	21.61	3.33E-06	5.15E-05	0.90	DLBCL_3
hsa-miR-3179	1.22	7.93	4.86E-03	2.82E-02	0.79	DLBCL_3
hsa-miR-20b-5p	1.22	26.31	2.90E-07	5.68E-06	0.99	DLBCL_3
hsa-miR-660-3p	1.24	17.14	3.47E-05	4.19E-04	0.84	DLBCL_3
hsa-miR-92b-5p	1.24	9.10	2.56E-03	1.66E-02	0.82	DLBCL_3
hsa-miR-7-1-3p	1.25	14.95	1.10E-04	1.20E-03	0.89	DLBCL_3
hsa-miR-326	1.26	15.78	7.10E-05	8.07E-04	0.83	DLBCL_3
hsa-miR-340-3p	1.27	13.39	2.52E-04	2.35E-03	0.84	DLBCL_3
hsa-miR-769-5p	1.28	12.37	4.37E-04	3.63E-03	0.82	DLBCL_3
hsa-miR-3158-3p	1.31	9.83	1.72E-03	1.21E-02	0.75	DLBCL_3
hsa-miR-576-3p	1.31	8.23	4.13E-03	2.49E-02	0.79	DLBCL_3
hsa-miR-186-5p	1.32	19.58	9.65E-06	1.34E-04	0.76	DLBCL_3
hsa-miR-1249	1.32	8.12	4.37E-03	2.58E-02	0.75	DLBCL_3
hsa-miR-598	1.32	16.48	4.91E-05	5.84E-04	0.91	DLBCL_3
hsa-miR-192-3p	1.33	13.51	2.38E-04	2.27E-03	0.79	DLBCL_3
hsa-miR-103a-3p	1.34	74.04	7.65E-18	1.97E-15	0.98	DLBCL_3
hsa-miR-941	1.35	10.37	1.28E-03	9.38E-03	0.84	DLBCL_3
hsa-miR-551b-3p	1.35	10.53	1.17E-03	8.72E-03	0.66	DLBCL_3
hsa-miR-26a-5p	1.35	38.87	4.53E-10	1.67E-08	1.00	DLBCL_3
hsa-miR-221-5p	1.37	14.13	1.71E-04	1.74E-03	0.80	DLBCL_3
hsa-miR-181a-3p	1.38	7.54	6.04E-03	3.34E-02	0.82	DLBCL_3
hsa-miR-188-5p	1.39	17.69	2.60E-05	3.47E-04	0.82	DLBCL_3
hsa-miR-330-5p	1.41	13.94	1.89E-04	1.87E-03	0.90	DLBCL_3

hsa-miR-4746-5p	1.41	7.08	7.78E-03	4.15E-02	0.67	DLBCL_3
hsa-miR-625-5p	1.42	31.62	1.87E-08	5.17E-07	0.99	DLBCL_3
hsa-miR-148b-5p	1.42	26.85	2.20E-07	4.73E-06	0.87	DLBCL_3
hsa-miR-222-3p	1.45	33.19	8.35E-09	2.48E-07	0.95	DLBCL_3
hsa-miR-1227	1.46	6.92	8.51E-03	4.45E-02	0.66	DLBCL_3
hsa-miR-30d-5p	1.46	27.73	1.39E-07	3.26E-06	0.84	DLBCL_3
hsa-miR-652-5p	1.46	21.30	3.93E-06	5.96E-05	0.91	DLBCL_3
hsa-miR-421	1.47	28.18	1.11E-07	2.67E-06	0.93	DLBCL_3
hsa-miR-324-5p	1.50	25.13	5.36E-07	9.63E-06	0.86	DLBCL_3
hsa-miR-125b-5p	1.52	13.16	2.87E-04	2.58E-03	0.80	DLBCL_3
hsa-miR-20a-3p	1.53	17.22	3.33E-05	4.19E-04	0.83	DLBCL_3
hsa-miR-501-5p	1.55	23.44	1.29E-06	2.23E-05	0.96	DLBCL_3
hsa-miR-425-5p	1.55	48.44	3.40E-12	1.75E-10	0.94	DLBCL_3
hsa-miR-3667-5p	1.58	13.33	2.61E-04	2.41E-03	0.76	DLBCL_3
hsa-miR-192-5p	1.58	56.37	6.00E-14	4.64E-12	0.99	DLBCL_3
hsa-miR-185-3p	1.59	22.65	1.94E-06	3.12E-05	0.90	DLBCL_3
hsa-miR-3130-5p	1.60	19.67	9.21E-06	1.32E-04	0.80	DLBCL_3
hsa-miR-339-5p	1.61	26.11	3.23E-07	6.08E-06	0.84	DLBCL_3
hsa-miR-641	1.61	12.81	3.44E-04	2.99E-03	0.77	DLBCL_3
hsa-miR-503	1.61	33.57	6.88E-09	2.13E-07	0.96	DLBCL_3
hsa-miR-33a-3p	1.65	17.15	3.45E-05	4.19E-04	0.94	DLBCL_3
hsa-miR-99a-5p	1.65	23.16	1.49E-06	2.50E-05	0.87	DLBCL_3
hsa-miR-500a-3p	1.65	39.92	2.64E-10	1.04E-08	0.92	DLBCL_3
hsa-miR-210	1.66	53.32	2.83E-13	1.99E-11	0.88	DLBCL_3
hsa-miR-589-3p	1.66	20.47	6.07E-06	8.85E-05	0.94	DLBCL_3
hsa-miR-502-3p	1.67	45.13	1.85E-11	8.40E-10	0.96	DLBCL_3
hsa-miR-1307-5p	1.69	15.71	7.38E-05	8.26E-04	0.80	DLBCL_3
hsa-miR-215	1.70	14.71	1.26E-04	1.33E-03	0.99	DLBCL_3
hsa-miR-378i	1.71	30.83	2.81E-08	7.50E-07	0.92	DLBCL_3
hsa-miR-181b-5p	1.74	38.32	5.99E-10	2.01E-08	0.94	DLBCL_3
hsa-miR-29c-5p	1.76	48.93	2.65E-12	1.46E-10	0.98	DLBCL_3
hsa-miR-3922-5p	1.76	9.20	2.41E-03	1.64E-02	0.67	DLBCL_3
hsa-miR-30a-5p	1.76	8.83	2.96E-03	1.89E-02	0.51	DLBCL_3
hsa-miR-378a-3p	1.77	45.62	1.44E-11	6.94E-10	0.99	DLBCL_3
hsa-miR-502-5p	1.82	26.39	2.79E-07	5.68E-06	0.96	DLBCL_3
hsa-miR-151a-3p	1.84	32.77	1.04E-08	2.97E-07	0.93	DLBCL_3
hsa-miR-194-5p	1.84	50.88	9.84E-13	5.85E-11	0.96	DLBCL_3
hsa-miR-29b-3p	1.84	28.73	8.33E-08	2.08E-06	0.95	DLBCL_3
hsa-miR-1537	1.90	12.47	4.13E-04	3.47E-03	0.86	DLBCL_3
hsa-miR-3074-3p	1.96	10.36	1.29E-03	9.38E-03	0.77	DLBCL_3
hsa-miR-3117-3p	1.98	10.14	1.45E-03	1.05E-02	0.32	DLBCL_3
hsa-miR-296-5p	2.15	27.60	1.49E-07	3.29E-06	0.95	DLBCL_3
hsa-miR-140-3p	2.16	56.65	5.19E-14	4.64E-12	0.96	DLBCL_3
hsa-miR-2278	2.17	23.06	1.57E-06	2.58E-05	0.89	DLBCL_3
hsa-miR-3176	2.21	7.54	6.02E-03	3.34E-02	0.77	DLBCL_3
hsa-miR-100-5p	2.23	15.50	8.25E-05	9.11E-04	0.78	DLBCL_3
hsa-miR-3688-3p	2.24	10.83	1.00E-03	7.58E-03	0.80	DLBCL_3
hsa-miR-4690-3p	2.25	18.03	2.18E-05	2.95E-04	0.73	DLBCL_3
hsa-miR-3909	2.27	56.87	4.65E-14	4.64E-12	0.98	DLBCL_3
hsa-miR-188-3p	2.31	13.48	2.41E-04	2.28E-03	0.79	DLBCL_3
hsa-miR-4714-3p	2.36	26.03	3.36E-07	6.18E-06	0.83	DLBCL_3

hsa-miR-378d	2.38	56.47	5.72E-14	4.64E-12	0.98	DLBCL_3
hsa-miR-362-5p	2.51	39.88	2.70E-10	1.04E-08	0.96	DLBCL_3
hsa-miR-378c	2.64	110.40	7.99E-26	3.09E-23	1.00	DLBCL_3
hsa-miR-1273c	2.65	38.57	5.27E-10	1.85E-08	0.92	DLBCL_3
hsa-miR-4454	2.72	50.95	9.45E-13	5.85E-11	0.91	DLBCL_3
hsa-miR-378f	2.83	33.95	5.66E-09	1.82E-07	0.83	DLBCL_3
hsa-miR-29c-3p	2.94	59.42	1.27E-14	1.97E-12	0.97	DLBCL_3
hsa-miR-4785	3.94	56.91	4.56E-14	4.64E-12	0.93	DLBCL_3
hsa-miR-124-3p	4.96	28.76	8.20E-08	2.08E-06	0.75	DLBCL_3
hsa-miR-34a-5p	5.35	132.02	1.48E-30	1.14E-27	0.97	DLBCL_3

Supplementary Table S3. Pathways influenced by 20 common dysregulated miRNAs in whole blood samples.			
KEGG pathway	p-value	#genes	#miRNAs
Adherens junction	1.91E-16	34	15
Cell cycle	1.12E-07	46	16
Proteoglycans in cancer	1.52E-06	50	15
Bacterial invasion of epithelial cells	2.34E-06	27	11
Bladder cancer	3.94E-06	21	14
Shigellosis	7.40E-06	26	12
Regulation of actin cytoskeleton	1.75E-05	56	15
Long-term depression	7.03E-05	17	13
p53 signaling pathway	8.13E-05	26	14
Glioma	8.13E-05	22	14
Hepatitis B	1.77E-04	38	15
Oocyte meiosis	3.18E-04	29	16
Colorectal cancer	4.23E-04	22	13
Melanoma	4.23E-04	22	14
Chronic myeloid leukemia	4.23E-04	23	14
Pancreatic cancer	6.18E-04	24	14
Lysine degradation	9.78E-04	14	9
Prostate cancer	1.63E-03	29	14
Viral carcinogenesis	2.13E-03	42	14
FoxO signaling pathway	2.13E-03	39	16
TGF-beta signaling pathway	2.14E-03	25	13
Sphingolipid signaling pathway	2.94E-03	33	14
Salmonella infection	3.54E-03	26	15
Biotin metabolism	4.74E-03	1	2
Endometrial cancer	6.21E-03	16	14
Pathways in cancer	6.86E-03	79	14
Hippo signaling pathway	7.65E-03	38	14
Focal adhesion	9.28E-03	51	15
Non-small cell lung cancer	1.72E-02	17	14
HTLV-I infection	2.20E-02	60	15
Axon guidance	2.35E-02	27	15

Protein processing in endoplasmic reticulum	2.93E-02	39	15
MAPK signaling pathway	3.27E-02	53	16
Thyroid cancer	3.87E-02	9	13
ErbB signaling pathw	4.66E-02	25	14

Supplementary Figure 1. Contribution of variables to each sPLS-DA component in c-miRNAs. The bars are coloured according to outcome for which variable has a maximal median value.

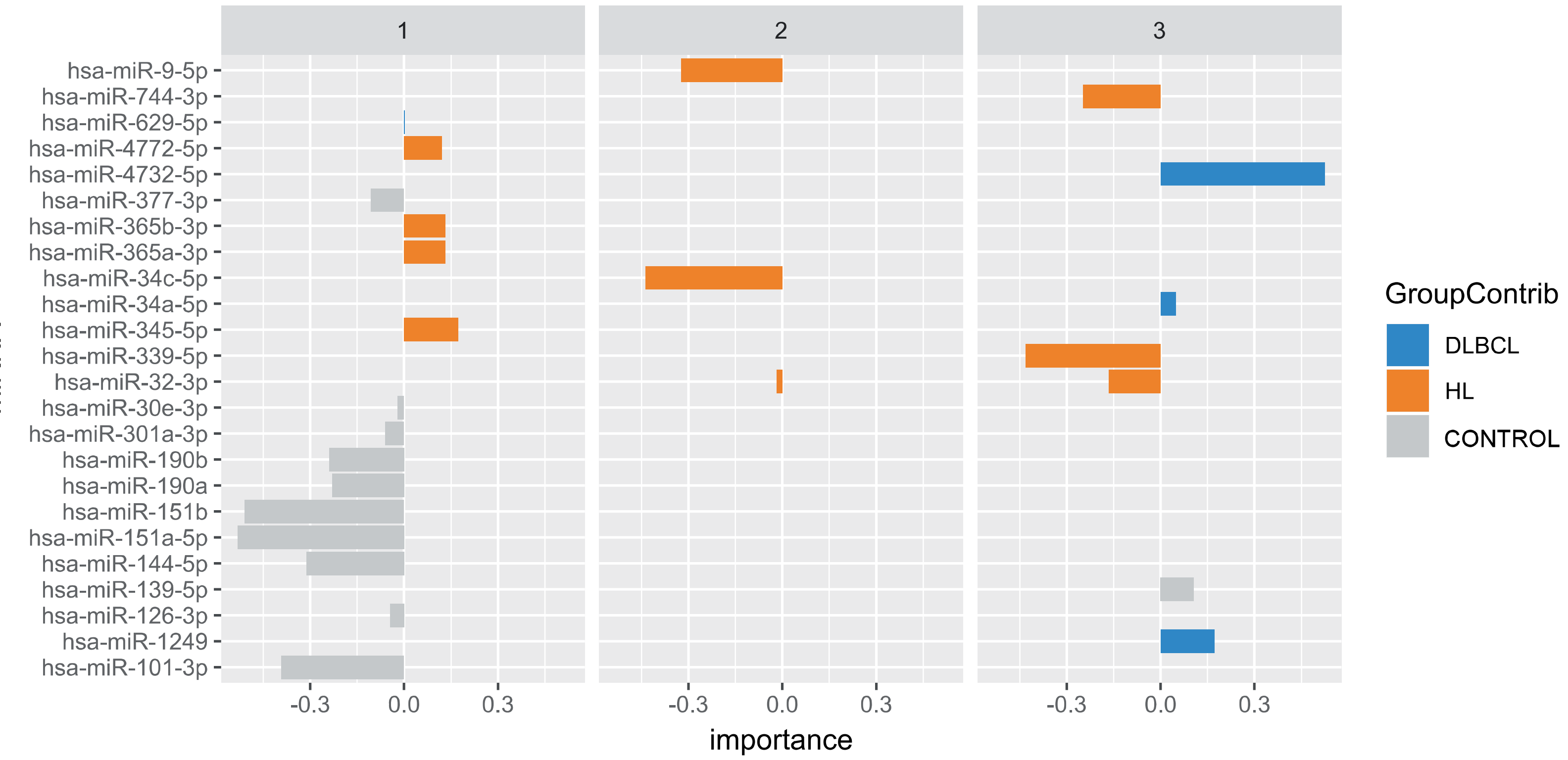

Supplementary Figure 2. Contribution of variables to each sPLS-DA component in wb-miRNAs. The bars are coloured according to outcome for which variable has a maximal median value.

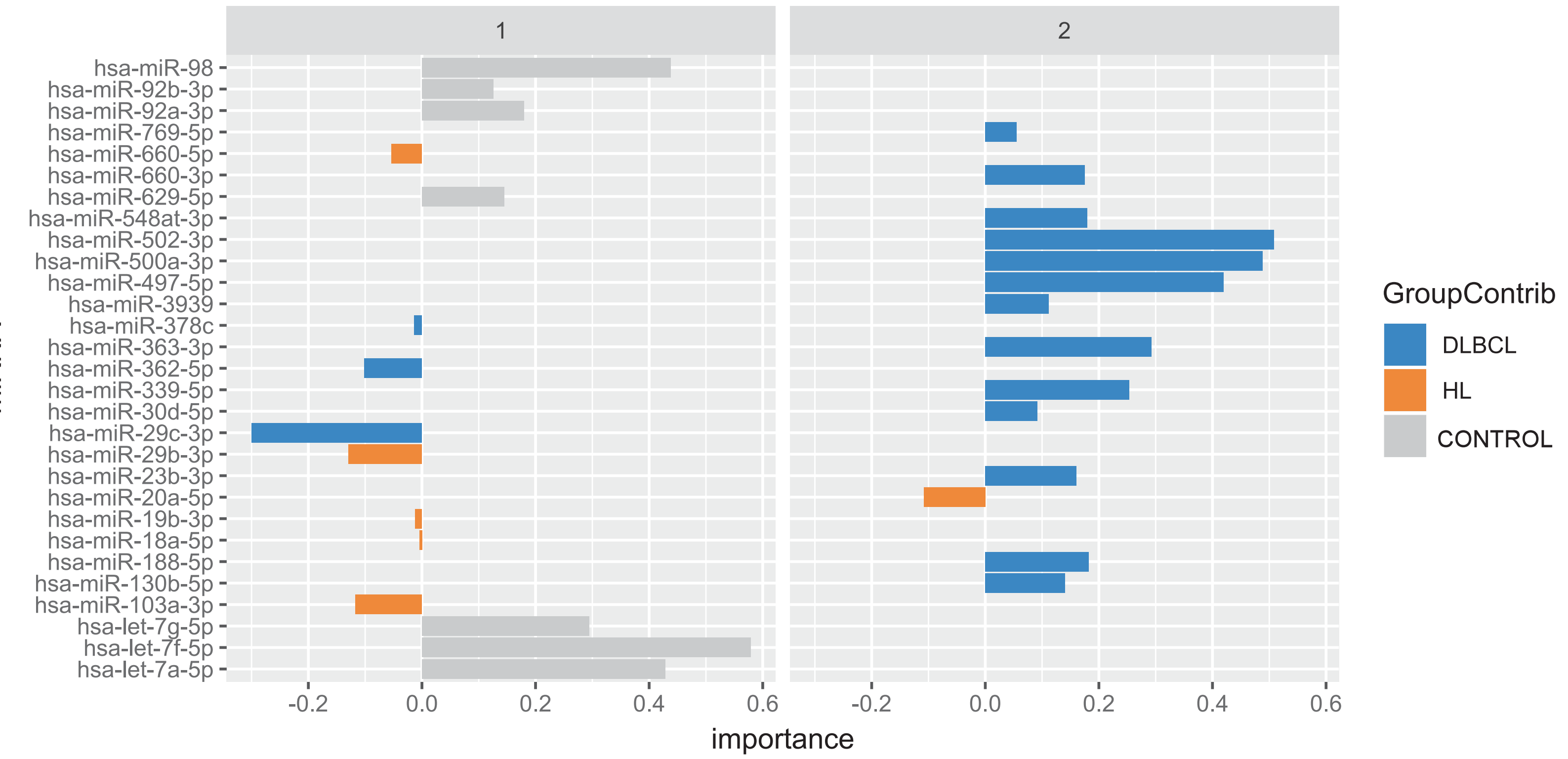

Supplement: Supplementary Materials — Supplementary Table S1: the results of differential expression test performed with edgeR for c-miRNAs, where logFC stands for fold-change logarithm, LR stands for likelihood ratio test statistic, PValue stands for P value in likelihood ratio test, QValue stands for P value after FDR correction, and AUC stands for area under the ROC curve. Supplementary Table S2: the results of differential expression test performed with edgeR for wb-miRNAs, where logFC stands for fold-change logarithm, LR stands for likelihood ratio test statistic, PValue stands for P value in likelihood ratio test, QValue stands for P value after FDR correction, and AUC stands for area under the ROC curve. Supplementary Table S3: pathways influenced by 20 common dysregulated miRNAs in whole blood samples. Supplementary Figure 1: contribution of variables to each sPLS-DA component in c-miRNAs. The bars are coloured according to outcome for which variable has a maximal median value. Supplementary Figure 2: contribution of variables to each sPLS-DA component in wb-miRNAs. The bars are coloured according to outcome for which variable has a maximal median value. [file 3212878.f1.pdf]
